# Cryptic Epoxytiglianes from the Kernels of the Blushwood Tree (*Fontainea picrosperma*)

**DOI:** 10.1021/acs.jnatprod.2c00226

**Published:** 2022-08-16

**Authors:** Giuseppina Chianese, Hawraz Ibrahim M. Amin, Chiara Maioli, Paul Reddell, Peter Parsons, Jason Cullen, Jenny Johns, Herlina Handoko, Glen Boyle, Giovanni Appendino, Orazio Taglialatela-Scafati, Simone Gaeta

**Affiliations:** † Dipartimento di Farmacia, Università di Napoli Federico II, Via Montesano 49, 80131 Napoli, Italy; ‡ Dipartimento di Scienze del Farmaco, Università del Piemonte Orientale, Largo Donegani 2, 28100 Novara, Italy; § QBiotics Group Limited, PO Box 166, Yungaburra, 4884, QLD, Australia; ∧ Drug Discovery Group, QIMR Berghofer Medical Research Institute, 300 Herston Road, Herston, 4006, QLD, Australia; + School of Biomedical Sciences, Faculty of Medicine, University of Queensland, Brisbane, 4072, QLD, Australia

## Abstract

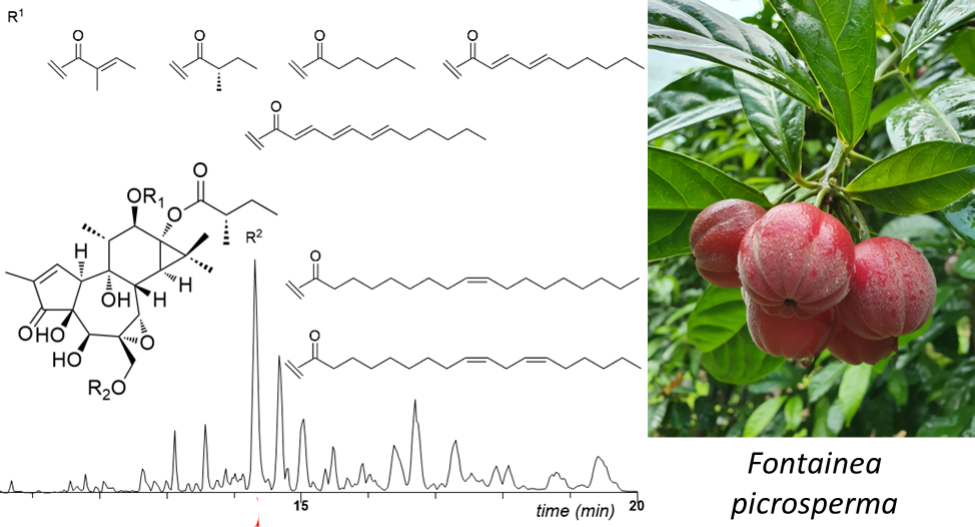

The kernels of the Australian blushwood tree (*Fontainea picrosperma*) are the source of the veterinary anticancer drug tigilanol tiglate (**2a**, Stelfonta) and contain a concentration of phorboids significantly higher than croton oil, the only abundant source of these compounds previously known. The oily matrix of the blushwood kernels is composed of free fatty acids and not by glycerides as found in croton oil. By active partitioning, it was therefore possible to recover and characterize for the first time a cryptic tigliane fraction, that is, the diterpenoid fraction that, because of its lipophilicity, could not be obtained by solvent partition of crude extracts. The cryptic tigliane fraction accounted for ca. 30% of the tigliane kernel titer and was quantified by ^1^H NMR spectroscopy and profiled by HPLC-MS. Long-chain (linoleates and/or oleates) 20-acyl derivatives of the epoxytigliane diesters tigilanol tiglate (EBC-46, **2a**), EBC-47 (**4a**), EBC-59 (**5a**), EBC-83 (**6a**), and EBC-177 (**7a**) were identified. By chemoselective acylation of EBC-46 (**2a**) and EBC-177 (**7a**) the natural triesters **2b** and **7b** and a selection of analogues were prepared to assist identification of the natural compounds. The presence of a free C-20 hydroxy group is a critical requirement for PKC activation by phorbol esters. The unexpected activity of 20-linoleoyl triester **2b** in a cytotoxicity assay based on PKC activation was found to be related mainly to its hydrolysis to tigilanol tiglate (**2a**) under the prolonged conditions of the assay, while other esters were inactive. Significant differences between the esterification profile of the epoxytigliane di- and triesters exist in *F. picrosperma*, suggesting a precise, yet elusive, blueprint of acyl decoration for the tigliane polyol 5-hydroxyepoxyphorbol.

In 1857, Bucheim reported the surprising observation that the nonirritant and tasteless ethanol-insoluble fraction of the obnoxious oil from the kernels of *Croton tiglium* L. (croton oil, Euphorbiaceae) became highly irritating and extremely sharp by mild treatment with bases.^1^ This puzzling observation was clarified mechanistically a century later, when it was discovered that the kernels of *C. tiglium*, and possibly also of other oleaginous sources of phorbol (**1a**), contain significant amounts of tigliane esters, which, due to an exceedingly high lipophilicity, cannot be recovered from their fatty matrix by direct extraction, but only after selective acidic transesterification to more hydrophilic 12,13-diesters.^2^ These cryptic compounds, estimated to contribute ca. 50% of the phorbol titer of croton oil,^3^ have been assumed to be 20-acyl derivatives of the diesters recovered from the lipid matrix by partition,^2,3^ a suggestion supported by reactivity studies on phorbol, chemotaxonomic evidence, and bioactivity considerations. Thus, transesterification experiments on phorbol pentaacetate showed that, within the possible deacylation sites, esters of the allylic C-20 hydroxy group and of the acyloin C-4-hydroxy have similar reactivity, with the C-9-, C-12-, and C-13-acetates being significantly less reactive.^4^ Since few phorbol 20-triesters have been isolated from euphorbiaceous lattices, a less lipophilic matrix than kernel oils,^5^ the C-20 hydroxy group was considered the most plausible additional acylation site in cryptic tiglianes. The modest activity of croton oil in assays of tumor promotion after removal of the phorbol diester fraction supports this view, since a free C-20 hydroxy group is required for positive results in this assay.^2^ On the other hand, comparison of the esterification profile of the native tigliane diesters obtained from partition with that of the semisynthetic diesters recovered after transesterification of the cryptic fraction showed differences in the acylation pattern of the C-12- and C-13-hydroxy substituents,^2^ suggesting a precise strategy of acyl decoration of the parent diterpene polyol. The nature of the C-20 acyl residue has remained elusive and was identified tentatively as a short-chain acid because of the sluggish reactivity of long-chain tigliane 20-esters in transesterification reactions.^6^

**Chart 1:**
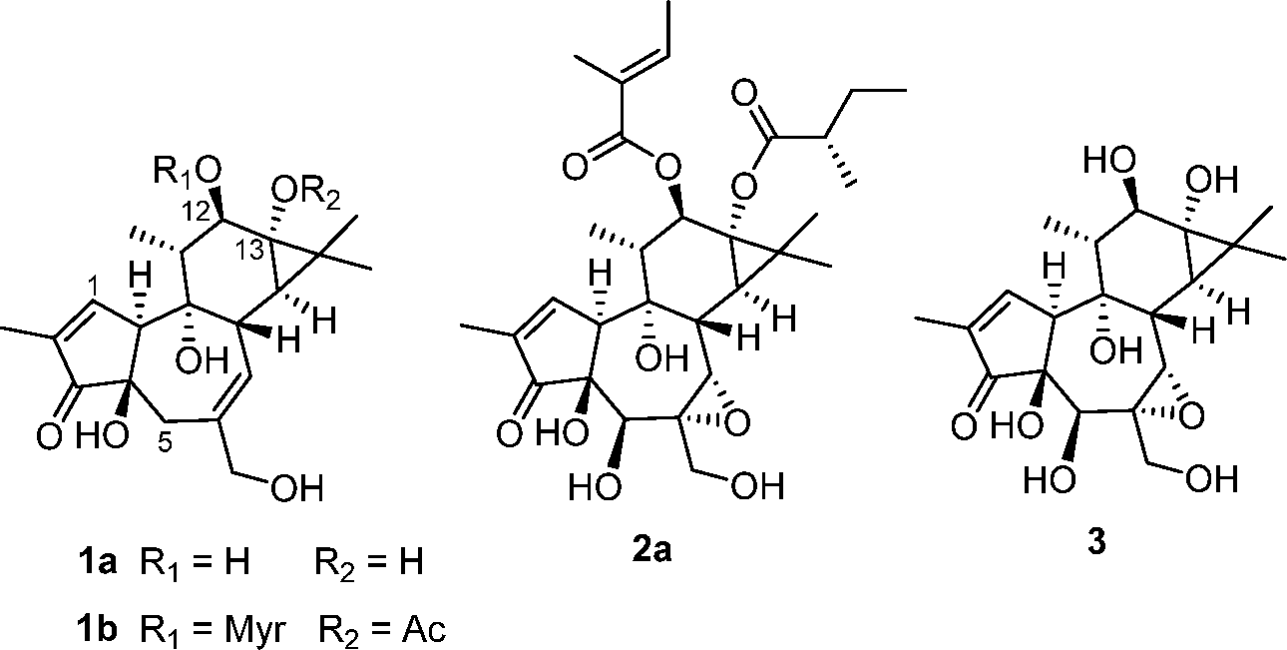


The kernels of *C. tiglium* have long remained the only abundant (ca. 1%) source of phorbol (**1a**), a diterpenoid polyol, of which its esters are trace constituents of many euphorbiaceous plants.^6^ A second abundant source of tiglianes was recently identified in the kernels of *Fontainea picrosperma* C. T. White (Euphorbiaceae), an endemic plant to the Australian rainforest and the source of the epoxytigliane veterinary anticancer drug tigilanol tiglate (**2a**).^7^ The kernels of this plant contain a much higher concentration of tigliane esters compared to croton oil (up to 7%). Additionally, these diterpenoids are almost exclusively based on a single polyol (5β-hydroxy-6α,7α-epoxyphorbol, **3**), while croton oil contains significant amounts also of 4-deoxyphorbol esters.^6^ In light of the surprising observation that the lipid matrix of the kernels from *F. picrosperma* is mostly made up of free fatty acids and not by triglycerides, the cryptic tigliane fraction could be purified after removal of the fatty acids with a basic partition. Its major constituents were identified, and their acylation pattern and bioactivity compared with those of the epoxytigliane diesters isolated from more polar fractions of kernel extracts.^7^

## Results and Discussion

**Figure 1:**
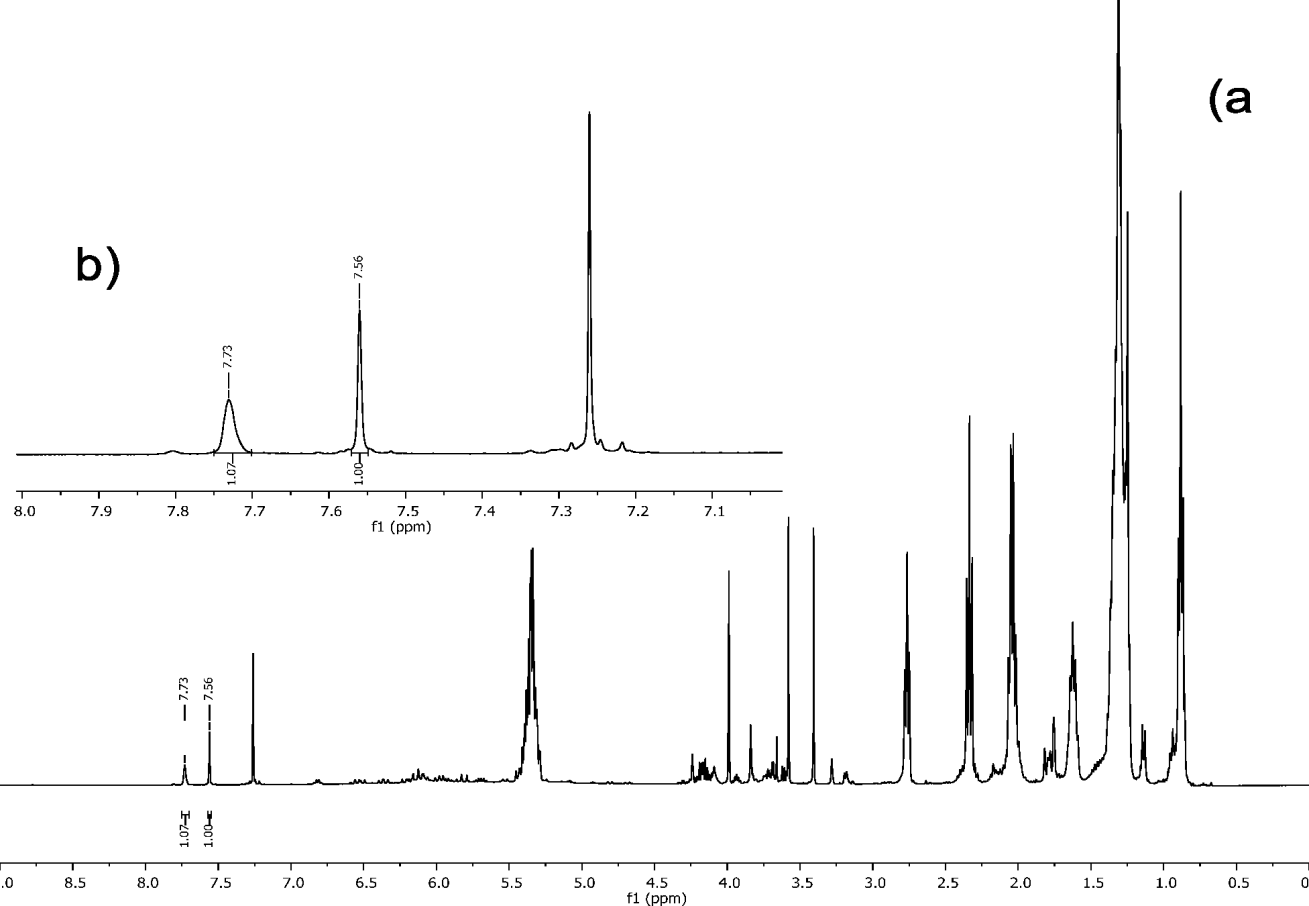
^1^H NMR spectrum of the lipophilic fraction of blushwood kernel oil. (a) Full spectrum. (b) Expansion of the downfield area.

A methanol extract from the kernels of *F. picrosperma* was partitioned between hexane and 80% methanol, recovering a diester fraction containing tigilanol tiglate (**2a**) as the major constituent. The concentration of the cryptic esters remaining in the lipophilic fraction could not be estimated by comparison of the isolation yield of 5β-hydroxy-6α,7α-epoxyphorbol (**3**) from the primary crude extract and from the partitioned extract, since, unlike phorbol (**1a**),^8^ this polyol proved unstable under basic transesterification conditions. Detection and quantitation of the cryptic esters were therefore conducted using ^1^H NMR spectroscopy.^9^ The presence of cryptic tigliane esters in the lipophilic phase was revealed by the detection of a downfield signal for the tigliane H-1 proton around δ 7.70 (Figure 1), a chemical shift not significantly affected by the acylation pattern of the diterpenoid core. Moreover, polar compounds like phenolics do not partition into highly lipophilic matrixes, and this region of the spectrum was free from interfering signals. The collective titer of tigliane esters in the lipophilic phase could therefore be assessed by quantitative ^1^H NMR, comparing the intensity of the tigliane H-1 signal with that of H-8 of caffeine (resonating at δ 7.56) as internal reference. Using the molecular weight of the major cryptic ester of tigilanol tiglate (the linoleate **2b**, see below), a value of 19.5% could be calculated, corresponding to ca. 35% of the total titer of tigliane triesters of the kernels (see the Experimental Section).

Surprisingly, along with the presence of tigliane triesters, the ^1^H NMR spectrum also revealed that the lipid matrix of the kernels was made up almost exclusively of fatty acids and not triglycerides (Figure 1), an unprecedented observation in oleaginous euphorbiaceous plants, but well documented in tropical palms such as saw palmetto [*Serenoa repens* (W. Bartram) Small].^10^ The fatty acids could be removed by liquid–liquid partition of the hexane phase with 2% KOH, a base selected because of the higher water solubility of the fatty acid potassium salts compared to the sodium salts.^11^ Alternatively, a solid–liquid partition based on filtration over neutral alumina could also be used, but the large load of alumina required made this quick and straightforward method unsuitable for large-scale preparations. The ^1^H NMR spectrum of the defatted triester fraction confirmed the location of the additional ester group at the C-20 hydroxy group, since the AB system of the C-20 oxymethylene protons had undergone a significant downfield shift compared to the corresponding diesters (Δδ ca. 0.50; Supporting Information, Figure S1).^7^ Remarkably, only minor amounts of residual diesters were present, since the integration of the downfield signal of H-20a of the triester was ca. 95% of the H-1 singlet, which is diagnostic of diesters and triesters. The close similarity between the various triesters and stability issues made their isolation impractical, and it was decided to profile the composition of the cryptic fraction by HPLC-MS. To aid identification and provide samples sufficiently pure for bioactivity evaluation, a selection of cryptic triesters was prepared from their corresponding diesters.

**Figure 2:**
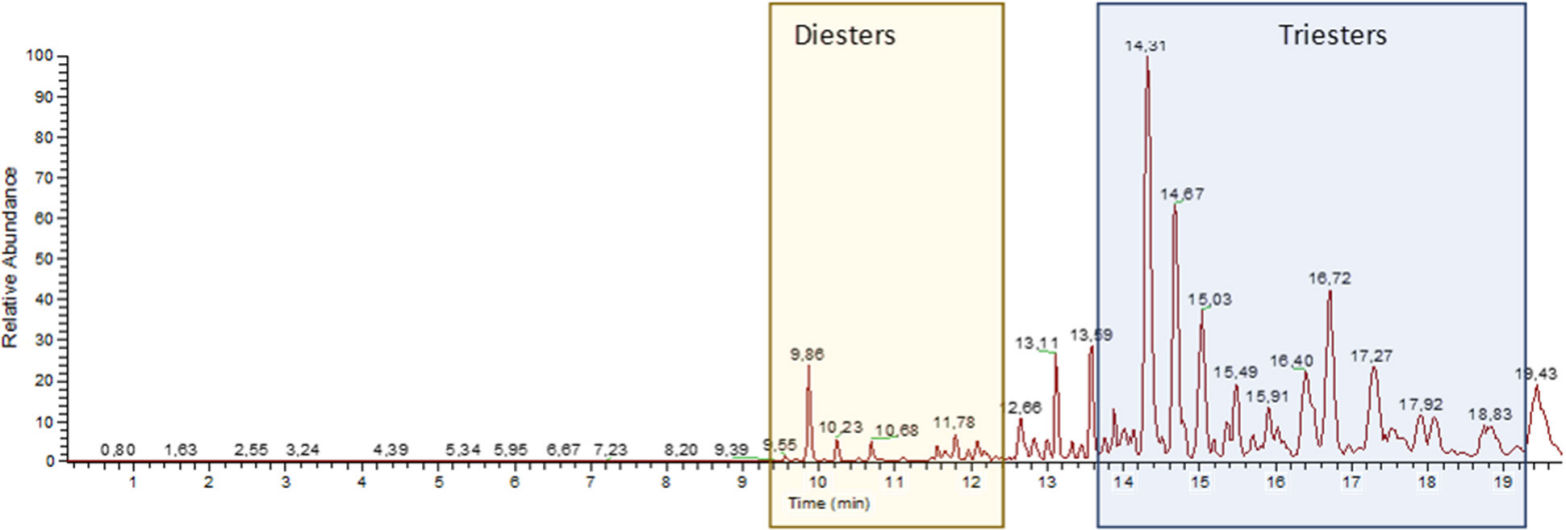
LC-MS chromatogram (positive-ion HRESI) of the defatted triester fraction.

The LC-HRMS and MS/MS analysis was carried out in the positive-ion mode on an LTQ-Orbitrap instrument using a RP18 stationary phase and a MeOH–water gradient. The base peak chromatogram obtained is shown in Figure 2. The analysis of the LC-HRMS base peak chromatogram allowed the identification of nine epoxytigliane triesters and five diesters, present in trace amounts, based on their HRMS, molecular formula analysis, and fragmentation pattern (Table 1).

**Table 1:** Annotation of the Major Peaks in the LC-MS Profile (Positive-Ion Mode) of the Defatted Triester Fractions of *Fontaineapicrosperma*

*t*_R_ (min)	[M + H]^*+*^ measured	molecular formula	Δppm	diagnostic fragments *m*/*z*	annotation
9.86	563.2852	C_30_H_43_O_10_	0.259	463 (100), 343 (10), 325 (8)^a^	EBC-46 (**2a**)
10.23	565.3016	C_30_H_45_O_10_	1.497	463 (100), 343 (8), 325 (8)	EBC-47 (**4a**)
10.68	579.3173	C_31_H_47_O_10_	1.529	463 (100), 343 (12), 325 (10)	EBC-83 (**6a**)
11.78	631.3486	C_35_H_51_O_10_	1.514	463 (100), 343 (8), 325 (7)	EBC-177 (**7a**)
12.06	657.3640	C_37_H_53_O_10_	0.952	463 (100), 343 (8), 325 (7)	EBC-59 (**5a**)
14.31	825.5157	C_48_H_73_O_11_	1.224	725 (10), 623 (43), 427 (75), 343 (75), 325 (100)	20-linoleyl-EBC46 (**2b**)^b^
14.67	827.5327	C_48_H_75_O_11_	1.922	725 (10), 623 (42), 427 (80), 343 (80), 325 (100)	20-linoleyl-EBC47 (**4b**)
15.03	841.5474	C_49_H_77_O_11_	1.569	725 (10), 623 (40), 427 (75), 343 (77), 325 (100)	20-linoleyl-EBC83 (**6b**)
15.49	829.5470	C_48_H_77_O_11_	1.158	727(10), 625 (55), 427 (77), 343 (77), 325 (100)	20-oleyl-EBC47 (**4c**)
15.91	843.5634	C_49_H_79_O_11_	1.968	727(17), 625 (55), 427 (77), 343 (75), 325 (100)	20-oleyl-EBC83 (**6c**)
16.72	893.5786	C_53_H_81_O_11_	1.422	725 (12), 623 (55), 427 (70), 343 (80), 325 (100)	20-linoleyl-EBC177 (**7b**)
17.27	919.5944	C_55_H_83_O_11_	1.555	725 (25), 623 (50), 427 (70), 343 (65), 325 (100)	20-linoleyl-EBC59 (**5b**)^b^
18.09	895.5955	C_53_H_83_O_11_	2.470	727(17), 625 (20), 427 (60), 343 (70), 325 (95)	20-oleyl-EBC177 (**7c**)^b^
18.83	921.6097	C_55_H_85_O_11_	1.194	727(12), 625 (25), 427 (45), 343 (40), 325 (65)	20-oleyl-EBC59 (**5c**)

a In brackets is the relative abundance of each diagnostic fragment peak.

b Compound identification supported by the comparison with the semisynthesis standards.

The first compounds eluted were the residual diester epoxytiglianes, characterized by the formation in the full MS scan of the diagnostic peak at *m*/*z* 463.2, identified as 13-(methylbutyryl)-5β-hydroxy-6α,7α-epoxyphorbol (C_25_H_35_O_8_ [F1 + H]^+^), originating from fragmentation with loss of the acyl group at C-12. Hence, the differences between the parent masses and the common fragment F1 provided information about the nature of the C-12 acyl groups, allowing the identification of EBC-46 (**2a**, C_30_H_42_O_10_), EBC-47 (**4a**, C_30_H_44_O_10_), EBC-83 (**6a**, C_31_H_46_O_10_), EBC-177 (**7a**, C_35_H_50_O_10_), and EBC-59 (**5a**, C_37_H_52_O_10_). Elution of the triesters started after 14 min, and they could be identified, following the same approach described previously, as 20-oleyl or 20-linoleyl derivatives of **2a**, **4a**, **5a**, **6a**, and **7a**.

**Chart 2:**
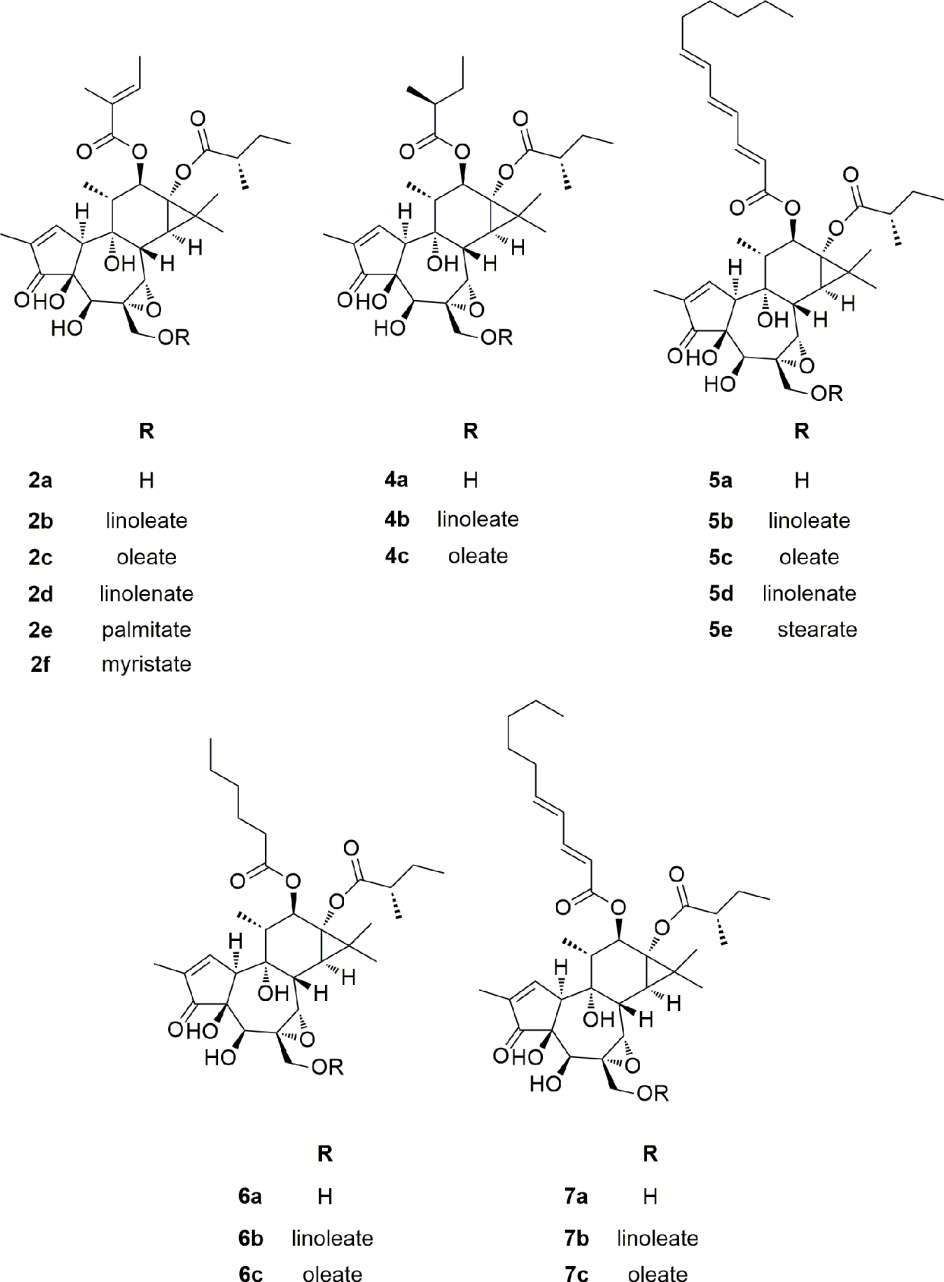


Notably, while the fatty acid fraction was made up almost exclusively by saturated fatty acids, the acyl group at C-20 was, conversely, unsaturated in all triesters characterized, showing that the decoration of the tigliane polyol is selective and does not simply depend on the kernel fatty acid pool. Identification was guided by the diagnostic fragment of the F1-type (loss of the C-12 acyl group) in the (+)-HRESIMS spectra at *m*/*z* 725.5 for the 20-linoleyl derivatives **2b**, **4b**, **5b**, **6b**, and **7b** and at *m*/*z* 727.5 for the 20-oleyl derivatives **4c**–**7c**, respectively. Moreover, analysis of MS/MS fragmentation patterns of the base peaks and of the F1 fragments, selected from the full scan spectra, afforded diagnostic fragments that supported the presence of a common 13-(α-methylbutyryl)-5β-hydroxy-6α,7α-epoxyphorbol backbone and different esterification pattern at C-12 and C-20. Figures S1–S3 (Supporting Information) exemplify the assignment of fragmentation peaks for **2b, 4b**, and **4c**.

**Figure 3:**
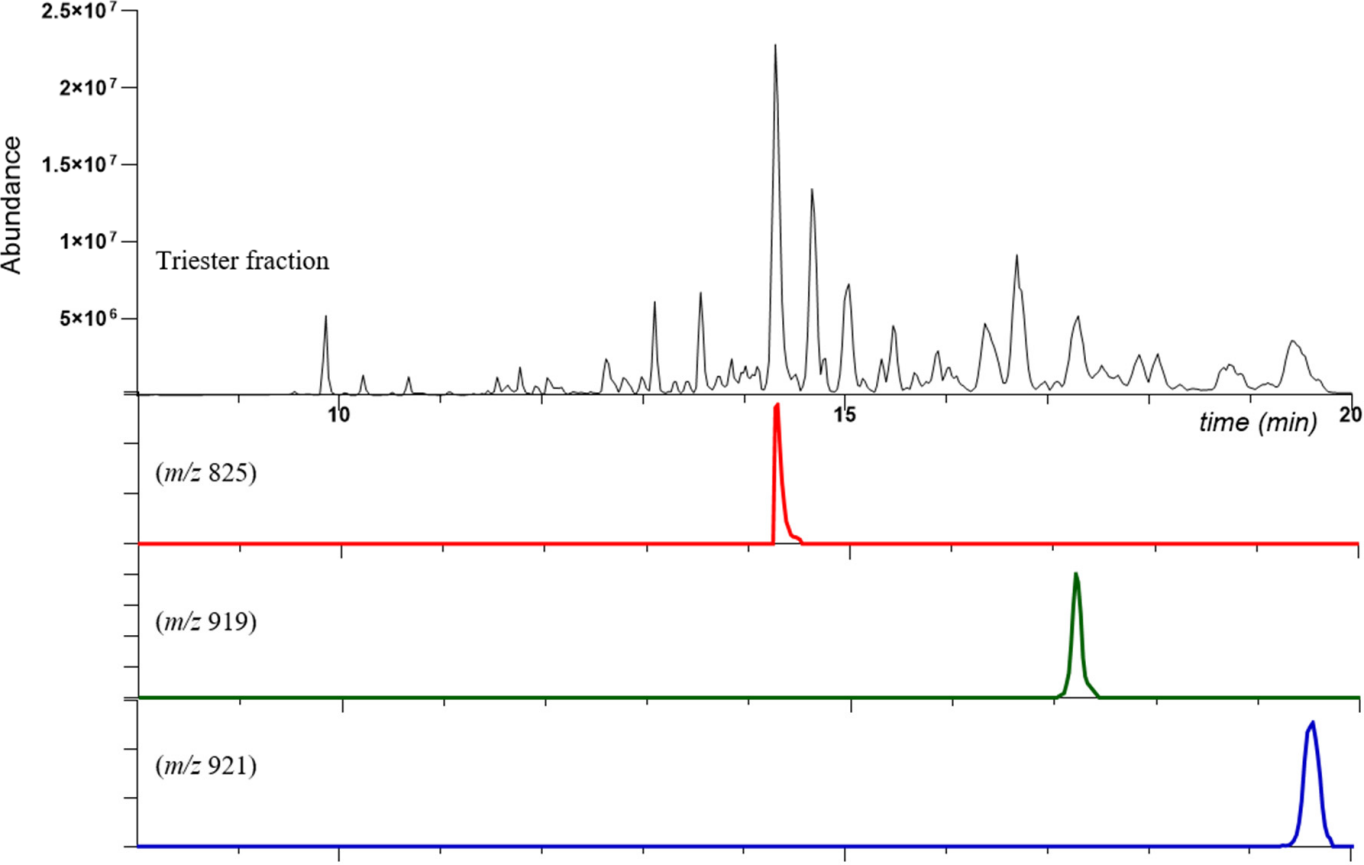
Stacked base peak chromatograms of samples and selected standards.

Epoxytigliane triesters were obtained by Steglich esterification of the two most abundant constituents of the native diester fraction, namely, tigilanol tiglate (EBC-46, **2a**) and EBC-59 (**5a**). In all cases, at complete conversion of the starting diester, variable amounts of the corresponding 5,20-tetraesters were also obtained, since the C-5 hydroxy group was only moderately less reactive than the C-20 hydroxy group. The availability of a library of synthetic triesters of EBC-46 and EBC-59 made it possible to confirm the assignments from LC-MS/MS analysis. Thus, the LC-MS profile of the *F. picrosperma* triester fraction was compared with the following nine synthetic standards analyzed in the same experimental conditions: linoleate, oleate, linolenate, and stearate esters at position C-20 of EBC-59 (**5b**–**5e**) and linoleate, oleate, linolenate, palmitate, and myristate esters (**2b**–**2f**) at position C-20 of EBC-46. Among them, only EBC-46-20-linoleate (**2b**), EBC-59-20-oleate (**5c**), and 20-linoleate (**5b**) showed the same retention time, thus confirming the assignment made (Figure 3). The absence of 20-oleyl EBC-46 (**2c**) in the natural mixture of epoxytigliane triesters was confirmed by the observation that an authentic synthetic standard of **2c** showed a different chromatographic behavior (*t*_R_ 14.94 min) compared to the other triesters, including the isobaric analogue **4b**.

Anticipating that **2a** might be released hydrolytically during bioassay, the activity of **2b** was compared with that of **2a** in a rapid (release of ROS from human neutrophils over 10 min) as well as in a long-term assay (inhibition of 7-day growth of the K562 PKC-sensitive leukemia cell line). The results (Table 2) showed that, in the rapid assay, the cryptic ester **2b** was inactive when compared with the prototypic PKC activator phorbol 12-myristate-13-acetate (PMA) and with tigilanol tiglate (**2a**), showing that a free C-20 hydroxy group is necessary for activity. On the other hand, in the long-term assay the linoleate **2b** showed an increased potency, presumably due to hydrolysis to **2a**.

**Table 2:** Production of ROS in Human Neutrophils (10 min Exposure) and Inhibition of K562 Growth (7-Day Incubation) of Phorbol 12-Myristate-13-acetate (**1b**, PMA) and the Epoxytiglianes **2a** and **2b**

compound	EC_50_ nM ROS production (95% Cl)	IC_50_ nM K562 growth inhibition (95% Cl)
**1b**	16.7 (13.2–21.2)	0.55 (0.51–0.58)
**2a**	125 (97.3–160.7)^7^	11.9 (10.8–13.3)^7^
**2b**	>3627	124 (55–280)

Consistent with observations on croton oil,^2,3^ the esterification pattern of tigliane tri- and diesters in the kernel oil from *F. picrosperma* was not found to be overlapping. The ester group at the C-13 hydroxy group was α-methylbutyric acid in both series of esters, but differences were evident in the nature of the acyl group bound to the C-12 hydroxy group. Thus, the C-12 α-methylbutyrate esters **4b** and **4c** were found as major constituents of the triester fraction, while their corresponding C-20 deacyl derivatives were only very minor constituents of the diester mixture,^7^ and differences also existed in the relative amounts of 12-dodecatrienoate and 12-decadienoate esters within the two series of compounds. Notably, the pattern of C-12 and C-20 acylation seems related, since the 20-linoleyl EBC46, a major constituent of the mixture, was not accompanied by its corresponding 20-oleate, while all the other 12-esters were. Acylation, a critical phenomenon to produce structural diversity,^12^ seems therefore highly controlled in *F. picrosperma*, with esterification of the C-20 hydroxy group being related to the nature of the C-12 ester group. Given the hydrolytic sensitivity of the C-20 ester group, it is tempting to speculate that acylation of the primary hydroxy group of phorboids is associated with the storage of lipase-sensitive cryptic bioactivity, in a strategy similar to the glucosidase-sensitive cryptic bioactivity of glucosinolates and cyanogenic glycosides.^13^

## Experimental Section

### General Experimental Procedures

Optical rotations (CHCl_3_) were measured at 589 nm on a P2000 (JASCO Europe s.r.l., Cremella, Italy) polarimeter. ^1^H and ^13^C NMR spectra were recorded with Bruker Avance NEO (400 MHz) and Bruker Avance NEO (500 MHz) spectrometers and referenced against solvent signals (^1^H NMR residual proton signal: CDCl_3_ δ 7.26; ^13^C NMR: CDCl_3_ δ 77.2). Multiplicities are abbreviated as follows: s (singlet), d (doublet), t (triplet) q (quartet), m (multiplet) br (broad signal). q-NMR measurements were carried out with a flip angle of 30° and a probe temperature of 298.0 K, setting the relaxation delay and the acquisition time at 1 and 94 s, respectively. NMR processing for all the samples (phase correction, baseline correction) and peak integrations were performed manually with the software MestReNova 11.0.3-18688. Thin-layer chromatography was performed with 0.2 mm precoated aluminum sheet TLC silica gel 60 F254 (Merck), visualized by UV inspection (254 nm), and stained with 5% methanolic H_2_SO_4_ and heating. Gravity column chromatography was carried out using Merck silica gel 60 (70–230 mesh) or Davisil (40–63 μm). The following chemicals were purchased from Fluorochem (Glossop, UK): linoleic acid, linolenic acid, EDC hydrochloride, and DMAP. Oleic acid, stearic acid, palmitic acid, and myristic acid were purchased from Sigma-Aldrich Chemie (Steinheim, Germany) and used without any further purification. Tigilanol tiglate (**2a**) was supplied by QBiotics Group Limited.

### Plant Material

*Fontainea picrosperma* kernels were collected in March 2020 from a QBiotics Group Limited plantation (Atherton Tablelands, QLD, Australia) and were identified by one of the authors (P.R.). A voucher specimen of the methanol extract from the kernels is deposited at the Dipartimento di Scienze del Farmaco, Università del Piemonte Orientale, Novara, Italy.

### Isolation of the Cryptic Tigliane Fraction

Frozen kernels (100 g) were extracted with acetone (500 mL × 2). Evaporation of the extract afforded a thick oil (31 g, 31%), which was partitioned between petroleum ether and 80% aqueous methanol. The higher lipophilic phase was evaporated, affording 20 g of a yellow oil, which was dissolved in petroleum ether (70 mL) and partitioned with 2% KOH in MeOH–H_2_O (80:20, 2 × 100 mL). The pooled petroleum ether fractions were dried, affording 3.2 g of a crude mixture of tigliane triesters as a yellow oil.

### Attempted Detection of **2b** in Kernels

The kernels of *F. picrosperma* (7.57 g) were minced and extracted with 33 mL of ethanol in ambient conditions for 24 h, and the supernatant was analyzed by HPLC on a Phenomenex Luna C_18_ column (150 mm × 4.6 mm, 3 μm) with a water–acetonitrile gradient. Semisynthetic **2b** (retention time 21 min) was used as the standard to verify its absence in the extract.

### Caffeine-Based q-^1^H NMR Analysis of the *F. picrosperma* Cryptic Tigliane Fraction

A sample (25.6 mg) of the lipophilic fraction of the primary extract was weighed into an NMR tube and diluted in 0.5 mL of CDCl_3_. The ^1^H NMR spectrum was recorded with 16 scans. Caffeine (1.1 mg, 98% purity) was added and the ^1^H spectrum was recorded under the same conditions. After spectral correction, the integration of the caffeine signal at δ 7.56 (H-8) was set at 1.0, corresponding to a 11.33 μM concentration, and the integrations of the other signal were calculated in terms of this reference value. The epoxytigliane signal at δ 7.73 ppm was calculated as 1.07, corresponding to a molar concentration of 12.12 μM, converted in weight % using the molecular weight of **2b** (824.51 g/mol), the most abundant EBC-46 triester. In this way, the concentration of epoxytigliane triesters in the lipophilic fraction was estimated as 19.5% (w/w). The overall calculation can be summarized as follows: Percentage of triesters in the sample = [molar concentration of caffeine × (integration of caffeine reference signal:integration of the trimester reference signal) × MW(**2b**)]:sample weight.^11^

### Esterification of Epoxytigliane Diesters

EDC·HCl (1.2 molar equiv) was added to a stirred 0.2 M solution of a carboxylic acid in CH_2_Cl_2_ at room temperature. After complete solubilization, a tigliane diester [EBC-46 (**2a**) or EBC-59 (**5a**)] and a catalytic amount of DMAP were sequentially added and stirring was continued overnight. The reaction mixture was then diluted with 10% aqueous Na_2_SO_4_ and extracted with CH_2_Cl_2_ (×3). The combined organic layers were dried over Na_2_SO_4_ and concentrated. Purification over silica gel (petroleum ether–ethyl acetate, 8:2 as eluant) afforded the corresponding 20-acyl triesters in 30–70% yield. Full spectroscopic data are reported for **2c**, **2d**, and **5e** as representative (yield: 67%, 46%, and 30%, respectively). The ^1^H NMR spectroscopic characterization details for **2b** and the remaining triesters are provided in the Supporting Information (Table S2).

#### 12-Tigloyl-13-(2-methylbutyryl)-5β-hydroxy-6α,7α-epoxyphorbol-20-oleate (**2c**):

colorless oil; [α]^25^_D_ −9.7 (*c* 1.3, CHCl_3_); ^1^H NMR (400 MHz, CDCl_3_) δ 7.72 (1H, dd, *J* = 2.7, 1.5 Hz, H-1), 6.82 (1H, dddd, *J* = 8.7, 7.2, 5.7, 1.6 Hz, H-3′), 5.97 (1H, brs, OH), 5.44 (1H, d, *J* = 9.9 Hz, H-12), 5.38–5.29 (2H, m, H-9‴ and H-10‴), 4.80 (1H d, *J* = 11.9 Hz, H-20a), 4.26 (1H, s, H-5), 4.10 (1H, t, *J* = 2.8 Hz, H-10), 3.82 (1H, d, *J* = 12.0 Hz, H-20b), 3.19 (1H, d, *J* = 6.6 Hz, H-8), 3.14 (1H, s, H-7), 2.39 (1H, sxt, *J* = 6.9 Hz, H-2″), 2.35 (2H, t, *J* = 6.9 Hz, H-2‴), 2.01 (4H, m, H-8‴ and H-11‴), 1.97 (1H, m, H-11), 1.82 (3H, t, *J* = 1.4 Hz, H-5′), 1.79 (3H, dd, *J* = 7.1, 1.3 Hz, H-4′), 1.76 (3H, dd, *J* = 2.9, 1.3 Hz, H-19), 1.73 (1H, m, H-3″b), 1.62 (4H, m, H-7‴ and H-12‴), 1.45 (1H, dq, *J* = 14.5, 7.3 Hz, H-3″a), 1.24–1.20 (24H), 1.14 (3H, d, *J* = 7.0 Hz, H-5″), 0.94 (3H, t, *J* = 7.4 Hz, H-4″), 0.86 (6H, overlapped, H-18 and H-18‴); ^13^C NMR (CDCl_3_, 125 MHz) δ 209.85 (C-3), 178.98 (C-1″), 173.60 (C-1‴), 167.56 (C-1′), 164.64 (C-1), 137.73 (C-3′), 133.53 (C-2), 130.13 (C-9‴), 129.90 (C-10‴), 128.59 (C-2′), 77.30 (C-9), 76.82 (C-12), 72.49 (C-4), 69.61 (C-5), 65.73 (C-20), 65.66 (C-13), 65.37 (C-7), 60.67 (C-6), 48.98 (C-10), 46.03 (C-11), 41.32 (C-2″), 36.32 (C-14), 35.98 (C-8), 34.29 (C-2‴), 32.05 (C-16‴), 29.92–29.26 (7C, oleyl chain), 27.37 (C-8‴), 27.33 (C-11‴), 26.76 (C-15), 26.31 (C-3″), 25.05 (C-3‴), 23.84 (C-17), 22.83 (C-17‴), 17.41 (C-16), 16.32 (C-5″), 15.22 (C-18), 14.59 (C-4′), 14.27 (C-18‴), 12.39 (C-5′), 11.77 (C-4″), 9.90 (C-19); (+)-HRESIMS *m*/*z* [M + H]^+^ 827.5311 (calcd for C_48_H_75_O_11_, 827.5309).

#### 12-Tigloyl-13-(2-methylbutyryl)-5β-hydroxy-6α,7α-epoxyphorbol-20-linolenate (**2d**):

colorless oil; [α]^25^_D_ −14.2 (*c* 0.85, CHCl_3_); ^1^H NMR (400 MHz, CDCl_3_) δ 7.72 (1H, dd, *J* = 2.7, 1.5 Hz, H-1), 6.82 (1H, qd, *J* = 6.3, 5.5, 2.9 Hz, H-3′), 5.97 (1H, brs, OH), 5.44 (1H, d, *J* = 9.9 Hz, H-12), 5.42–5.28 (6H, m, H-9‴, H-10‴, H-12‴, H-13‴, H-15‴, H-16‴), 4.80 (1H d, *J* = 11.9 Hz, H-20a), 4.26 (1H, s, H-5), 4.10 (1H, t, *J* = 2.7 Hz, H-10), 3.82 (1H d, *J* = 12.0 Hz, H-20b), 3.19 (1H, d, *J* = 6.5 Hz, H-8), 3.14 (1H, s, H-7), 2.84–2.77 (4H, m, H-11‴, H-14‴), 2.39 (1H, sxt, *J* = 6.9 Hz, H-2″), 2.34 (2H, t, *J* = 7.5 Hz, H-2‴), 2.06 (4H, m, H-8‴ and H-17‴), 1.96 (1H, dd, *J* = 10.0, 6.4 Hz, H-11), 1.82 (3H, t, *J* = 1.3 Hz,H-5′), 1.79 (3H, dd, *J* = 7.1, 1.3 Hz, H-4′), 1.76 (1H, dd, *J* = 2.9, 1.3 Hz, H-19), 1.73 (1H, m, H-3″b), 1.46 (1H, m, H-3″a), 1.32 (10H), 1.28 (3H, s, H-16), 1.25 (3H, s, H-17), 1.14 (3H, d, *J* = 7.0 Hz, H-5″), 0.97 (3H, t, *J* = 7.6 Hz, H-4″), 0.94 (3H, t, *J* = 7.4 Hz, H-18‴), 0.87 (3H, dd, *J* = 6.4 Hz, H-18). ^13^C NMR (CDCl_3_, 125 MHz) δ 209.86 (C-3), 178.99 (C-1″), 173.59 (C-1‴), 167.56 (C-1′), 164.64 (C-1), 137.74 (C-3′), 133.53 (C-2), 132.11 (C-16‴), 130.43 (C-9‴), 128.60 (C-2′), 128.44 (C-15‴), 128.42 (C-13‴), 127.87 (C-12‴), 127.28 (C-10‴), 77.30 (C-9), 76.82 (C-12), 72.49 (C-4), 69.61 (C-5), 65.75 (C-20), 65.67 (C-13), 65.58 (C-7), 60.67 (C-6), 48.99 (C-10), 46.04 (C-11), 41.33 (C-2″), 36.32 (C-14), 35.99 (C-8), 34.28 (C-2‴), 29.86–29.25 (4C, C-4‴, C-5‴, C-6‴, C-7‴), 27.37 (C-8‴), 26.76 (C-15), 26.32 (C-3″), 25.78 (C-11‴), 25.69 (C-14‴), 25.05 (C-3‴), 23.84 (C-17), 20.71 (C-17‴), 17.41 (C-16), 16.32 (C-5″), 15.22 (C-18), 14.59 (C-4′), 14.33 (C-18‴), 12.39 (C-5′), 11.77 (C-4″), 9.91 (C-19); (+)-HRESIMS *m*/*z* [M + H]^+^ 823.5001 (calcd for C_48_H_71_O_11_, 823.4996).

#### 12-Dodeca-2*E*,4*E*,6*E*-trienoyl-13-(2-methylbutyryl)-5β-hydroxy-6α,7α-epoxyphorbol-20-stearate (**5e**):

colorless oil; [α]^25^_D_ −8.1 (*c* 0.75, CHCl_3_); ^1^H NMR (400 MHz, CDCl_3_) δ7.72(1H, t, *J* = 1.5 Hz, H-1), 7.25 (1H, dd, *J* = 16.0, 11.3 Hz, H-3′), 6.53 (1H, dd, *J* = 15.0, 10.3 Hz, H-5′), 6.21 (1H, dd, *J* = 14.9, 11.3 Hz, H-4′), 6.17–6.05 (1H, m, H-6′), 5.98–5.90 (1H, overlapped, H-2′), 5.81 (1H, d, *J* = 15.2 Hz, H-7′), 5.44 (1H, d, *J* = 9.9 Hz, H-12), 4.80 (1H d, *J* = 11.9 Hz, H-20a), 4.26 (1H, s, H-5), 4.10 (1H, t, *J* = 3.1 Hz,H-10), 3.83 (1H d, *J* = 11.9 Hz, H-20b), 3.70 (1H, s, OH), 3.65 (1H, s, OH), 3.19 (1H, d, *J* = 7.0 Hz, H-8), 3.14 (1H, s, H-7), 2.40 (1H, sxt, *J* = 6.9 Hz, H-2″), 2.39 (2H, t, *J* = 6.9 Hz, H-2‴), 2.15 (2H, m, H-8′), 1.97 (1H, dd, *J* = 9.9, 6.5 Hz, H-11), 1.76 (1H, dd, *J* = 2.9, 1.3 Hz, H-19), 1.73 (1H, m, H-3″b), 1.60 (4H, overlapped, H-9′, H-3‴), 1.44 (1H, m, H-3″a), 1.26 (17H, overlapped, H-14, H-16, H-17, H-10′, H-11′, H-4‴ to H-15‴), 1.14 (3H, d, *J* = 7.1 Hz, H-5″), 0.95 (3H, t, *J* = 7.4 Hz, H-4″), (0.87 (9H, overlapped, H-18, H-12′, H-16‴); ^13^C NMR (CDCl_3_, 100 MHz) δ 209.87 (C-3), 179.02 (C-1″), 173.64 (C-1‴), 166.70 (C-1′), 164.63 (C-1), 145.46 (C-3′), 141.80 (C-5′), 141.24 (C-7′), 133.51 (C-2), 129.86 (C-6′), 127.68 (C-4′), 119.63 (C-2′), 77.29 (C-12), 76.71 (C-9), 72.55 (C-4), 69.47 (C-5), 65.73 (C-13), 65.60 (C-7), 65.48 (C-20), 60.72 (C-6), 48.94 (C-10), 45.93 (C-11), 41.33 (C-2″), 36.29 (C-14), 35.90 (C-8), 34.28 (C-2‴), 33.99, 33.10 (C-8′), 32.06 (C-16‴), 31.52 (C-10′), 29.83 (C-6‴), 29.79 (C-7‴), 29.76 (C-8‴), 29.73 (C-9‴), 29.61 (C-10‴), 29.58 (C-11‴), 29.49 (C-12‴), 29.42 (C-13‴), 29.39 (C-14‴), 29.27 (C-5‴), 29.22 (C-4‴), 28.75 (C-9′), 26.81 (C-15), 26.29 (C-3″), 25.04 (C-3‴), 24.86 (C-15‴), 23.80 (C-16), 22.82 (C-17‴), 22.62 (C-11′), 17.31 (C-17), 16.31 (C-5″), 15.22 (C-18), 14.25, 14.14 (C-12′), 14.12 (C-18‴), 11.76 (C-3″), 9.89 (C-19); (+)-HRESIMS *m*/*z* [M + H]^+^ 895.5931 (calcd for C_53_H_83_O_11_, 895.5935).

### LC-MS/MS Analysis

A Thermo LTQ Orbitrap XL mass spectrometer (Thermo Fisher Scientific Spa, Rodano, Italy) equipped with electrospray ion (ESI) MAX source coupled to a Thermo U3000 HPLC system (Agilent Technology, Cernusco sul Naviglio, Italy) was used. HPLC separation were performed on a Kinetex 2.6 μm polar C_18_ 100 Å (100 × 3 mm) column with a flow rate of 0.5 mL/min and the following eluent gradient: H_2_O (0.1% HCOOH)–MeOH: *t* = 0 min (1:1), *t* = 3 min (1:1), *t* = 10 min (5:95), *t* = 25 min (5:95), *t* = 25.5 min (1:1). The HRMS and MS_*n*_ spectra, in the positive mode, were recorded in data-dependent acquisition mode inducing fragmentation of the most intense peak for each scan. Source conditions were as follows: spray voltage, 4.8 kV; capillary voltage, 31 V; auxiliary gas, 15 (arbitrary units); sheath gas, 32; capillary temperature, 285 °C; normalized collision energy, 30; isolation width, 2.0; activation Q, 0.250; activation time, 30 ms. The acquisition range was *m*/*z* 250–2000.

### Cytotoxicity Evaluation

K562 leukemia cells (3 × 10^3^ cells per well) were seeded into U-bottom 96-well plates (Corning #3799) in 90 μL of medium. Serial dilution of compounds in the medium (to 10× final assay concentration) and 10 μL of compound/vehicle dilutions were added subsequently to cells in duplicate.^14^ Vehicle (ethanol)-only controls were prepared in a similar manner. Treated cells were then incubated in a humidified incubator at 37 °C, 5% CO_2_ for 7 days, and growth/viability was evaluated, according to the manufacturer’s instructions, with the CellTiter 96 AQueous One Solution cell proliferation assay kit (Promega). Absorbance values at 490 nm were measured using a H4 Hybrid Synergy plate reader (Biotek), with a medium-only control used for background subtraction. Modified absorbance values from each compound-treated well were then normalized to vehicle-treated samples, assessing the percent growth/survival in each sample. Percentage growth/survival was plotted against log_10_[compound] nM to generate absolute IC_50_ curves for using Prism v8.2.1 (GraphPad).

### Reactive Oxygen Species Production in PMN Cells

Polymorphonuclear neutrophil (PMN) cells were isolated from peripheral blood acquired from healthy volunteers via dextran sedimentation.^14^ Following isolation, PMN cells were resuspended in Hank’s balanced salt solution (HBSS) (containing CaCl_2_, MgCl_2_), 2% fetal calf serum (FCS), and 100 000 unstained cells inserted in duplicate into the wells of a black, half-volume 96-well plate (Greiner #675077) as controls for background fluorescence. The remaining PMN cells were stained subsequently with dihydroethidium (DHE; 5 μg/mL final concentration), and 100 000 cells were plated per well in the same half-area 96-well plate. Serial dilutions of PMA and the compounds under investigation were also compiled in HBSS and 2% FCS, after which these dilutions were added to the required wells in duplicate. Dihydroethidium fluorescence (ex: 485/20 nm; em: 620/40 nm) was measured in each well after 10 min of incubation (37 °C, 5% CO_2_) using a Hybrid H4 Synergy plate reader (BioTek). Fluorescence values (after background fluorescence subtraction) were converted to mean % reactive oxygen species (ROS) production values (based on the maximal DHE fluorescence signal from PMA-treated samples). Mean % ROS production values ± SD were plotted versus log10[compound], and absolute EC_50_ values and 95% confidence intervals determined using a custom algorithm (PRISM 8.0).

## Supporting Information Available

^1^H NMR data for the triesters **2a**,**b**, **2e**,**f**, and **5a**–**d**, original ^1^H NMR spectrum of the defatted lipidic fraction of *F. picrosperma* kernel extract, and ^1^H NMR, ^13^C NMR, and MS spectra of the compounds (PDF)
