# Supplementary material for: Cryptic Epoxytiglianes from the Kernels of the Blushwood Tree (Fontainea picrosperma)
Source: J Nat Prod. 2022 Aug 16;85(8):1959–66. doi: 10.1021/acs.jnatprod.2c00226 (PMC9425429; doi:10.1021/acs.jnatprod.2c00226)
Supplement: Supplementary file 1 — np2c00226_si_001.pdf [file np2c00226_si_001.pdf]

Cryptic Epoxytiglianes from the Kernels of the Blushwood Tree (*Fontainea picrosperma*)

Giuseppina Chianese, † Hawraz I. M. Amin, ‡ Chiara Maioli, ‡, Paul Reddell, § Peter Parsons, ^ Jason Cullen, ^+ Jenny Johns, ^ Herlina Handoko, ^ Glen Boyle, ^+ Giovanni Appendino, ‡ Orazio Taglialatela-Scafati, † and Simone Gaeta *,‡§

† Dipartimento di Farmacia, Università di Napoli Federico II, Via Montesano 49, 80131 Napoli, Italy

‡ Dipartimento di Scienze del Farmaco, Università del Piemonte Orientale, Largo Donegani 2, 28100 Novara, Italy

§ QBiotics Group Limited, PO Box 166, Yungaburra, 4884, QLD, Australia

^ Drug Discovery Group, QIMR Berghofer Medical Research Institute, 300 Herston Road, Herston, 4006, QLD, Australia

+School of Biomedical Sciences, Faculty of Medicine, University of Queensland, Brisbane, QLD, Australia

SUPPORTING INFORMATION

Index

Figure S1.1 - ^{1}H NMR spectrum of *F. picrosperma* seed kernels acetonic extract 4

Figure S1.2 - ^{1}H NMR spectrum of the defatted *F. picrosperma* seed kernels extract by liquid/liquid extraction 5

Figure S1.3 - ^{1}H NMR spectrum of the defatted *F. picrosperma* seed kernels extract by solid/liquid extraction....6

Figure S2 – a) (+)-HRESIMS spectra of **2a**, *m/z* 563.2852 $[M+H]^+$ b) (+)-HRESIMS/MS spectra of **2a** 7

Figure S3 - a) (+)-HRESIMS spectra of **2b**, *m/z* 825.5157 $[M+H]^+$ b) (+)-HRESIMS/MS spectra of **2b**, *m/z* 825.51 $[M+H]^+$ (upper), and fragment F1, *m/z* 725.5 $[M+H]^+$ (lower) 8

Figure S4 – a) (+)-HRESIMS spectra of **4a**, *m/z* 565.3016 $[M+H]^+$ b) (+)-HRESIMS/MS spectra of **4a** 10

a) 10

Figure S5 - a) (+)-HRESIMS spectra of **4b**, *m/z* 827.5327 $[M+H]^+$ b) (+)-HRESIMS/MS spectra of **4b**, *m/z* 827.53 $[M+H]^+$ (upper), and fragment F1, *m/z* 725.5 $[M+H]^+$ (lower) 11

Figure S6 - a) (+)-HRESIMS spectra of **4c**, *m/z* 829.5470 $[M+H]^+$ b) (+)-HRESIMS/MS spectra of **4c**, *m/z* 829.55 $[M+H]^+$ (upper), and fragment F1, *m/z* 727.5 $[M+H]^+$ (lower) 13

b) 14

Figure S7 – a) (+)-HRESIMS spectra of **5a**, *m/z* 657.3640 $[M+H]^+$ b) (+)-HRESIMS/MS spectra of **5a**....15

Figure S8 – a) (+)-HRESIMS spectra of **5b**, *m/z* 919.5944 $[M+H]^+$ b) (+)-HRESIMS/MS spectra of **5b** 16

Figure S9 – a**)** (+)-HRESIMS spectra of **5c**, *m/z* 921.6097 $[M+H]^+$ b) (+)-HRESIMS/MS spectra of **5c** 17

Figure S10 – a) (+)-HRESIMS spectra of **6a**, *m/z* 579.3173 $[M+H]^+$ b) (+)-HRESIMS/MS spectra of **6a** 18

Figure S11 – a) (+)-HRESIMS spectra of **6b**, *m/z* 841.5474 $[M+H]^+$ b) (+)-HRESIMS/MS spectra of **6b** 19

Figure S12 – a) (+)-HRESIMS spectra of **6c**, *m/z* 843.5634 $[M+H]^+$ b) (+)-HRESIMS/MS spectra of **6c** 20

Figure S13 – a) (+)-HRESIMS spectra of **7a**, *m/z* 631.3486 $[M+H]^+$ b) (+)-HRESIMS/MS spectra of **7a**....21

Figure S14 – a) (+)-HRESIMS spectra of **7b**, *m/z* 893.5786 $[M+H]^+$ b) (+)-HRESIMS/MS spectra of **7b**....22

Figure S15 – a) (+)-HRESIMS spectra of **7c**, *m/z* 895.5955 $[M+H]^+$ b) b) (+)-HRESIMS/MS spectra of **7c**....23

Figure S16.1 - 24

Figure S17.1 - 24

Figure S18.1 ^{1}H NMR spectrum of 12-tigloyl-13-(2-methylbutyryl)-5β-hydroxy-6α,7α -epoxyphorbol-20-oleate, **2c** (400 MHz, $CDCl_3$)....25

Figure S18.2 – ^{13}C NMR spectrum of 12-tigloyl-13-(2-methylbutyryl)-5β-hydroxy-6α,7α -epoxyphorbol-20-oleate, **2c** (125 MHz, $CDCl_3$)....25

Figure S19.1 - ^{1}H NMR spectrum of 12-tigloyl-13-(2-methylbutyryl)-5β-hydroxy-6α,7α -epoxyphorbol-20-linolenate, **2d** (400 MHz, $CDCl_3$) 26

Figure S19.2 - ^{13}C NMR spectrum of 12-tigloyl-13-(2-methylbutyryl)-5β-hydroxy-6α,7α -epoxyphorbol-20-linolenate, **2d** (125 MHz, $CDCl_3$) 26

Figure S20.1 – ^{1}H NMR spectrum of 12-(2*E*,4*E*,6*E*)-dodecatrienoyl-13-(2-methylbutyryl)-5β-hydroxy-6α,7α-epoxyphorbol -20-stearate, **5e** (400 MHz, $CDCl_3$)....27

Figure S20.2 – ^{1}H NMR spectrum of 12-(2*E*,4*E*,6*E*)-dodecatrienoyl-13-(2-methylbutyryl)-5β-hydroxy-6α,7α-epoxyphorbol -20-stearate, **5e** (100 MHz, $CDCl_3$)....27

Table S2 – ^{1}H NMR data for the semisynthetic epoxytigliane triesters and related starting material (400 MHz, $CDCl_3$) 28

Figure S1.1 - ^{1}H NMR spectrum of *F. picrosperma* seed kernels acetonic extract

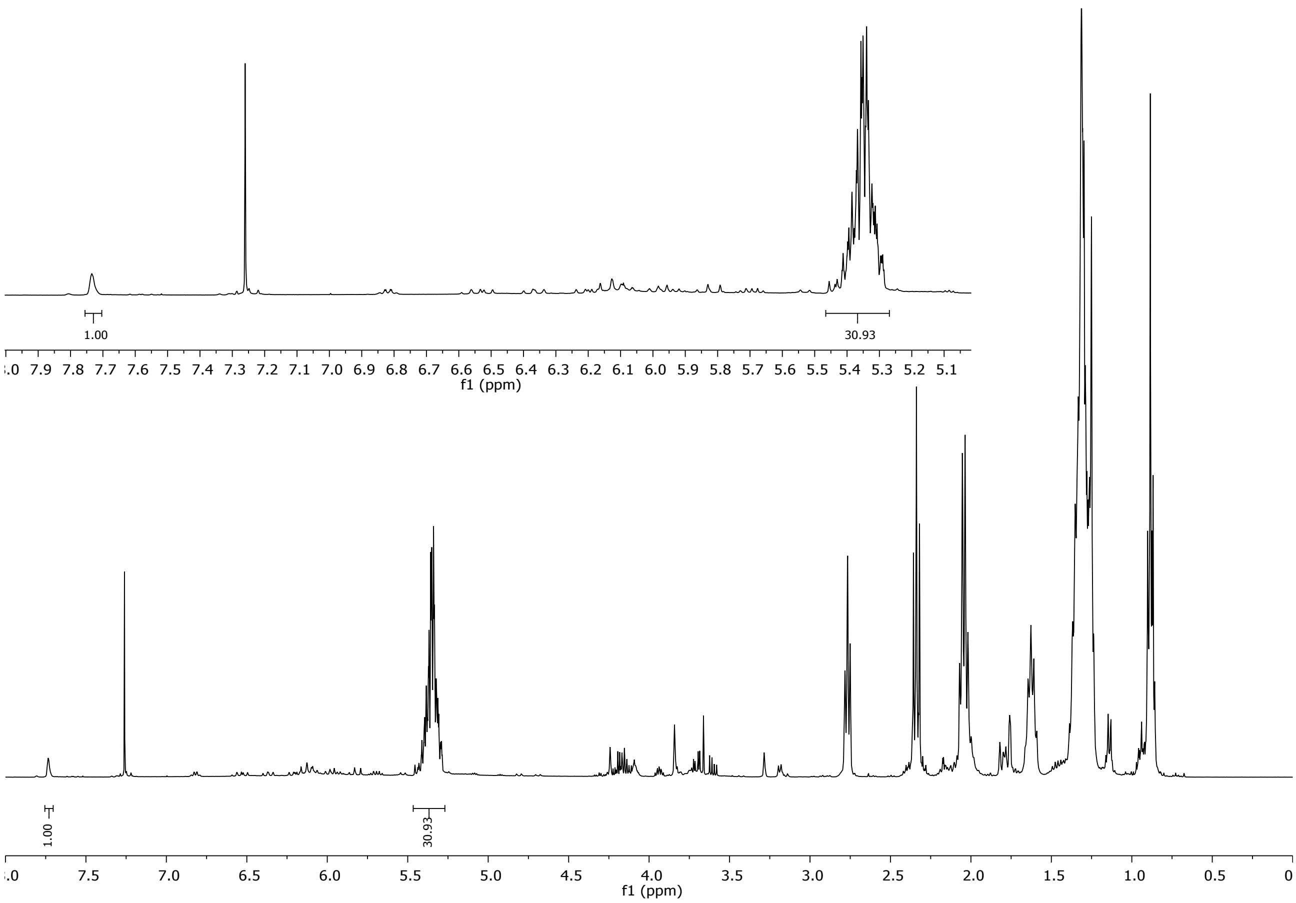

Figure S1.2 - ^{1}H NMR spectrum of the defatted *F. picrosperma* seed kernels extract by liquid/liquid extraction

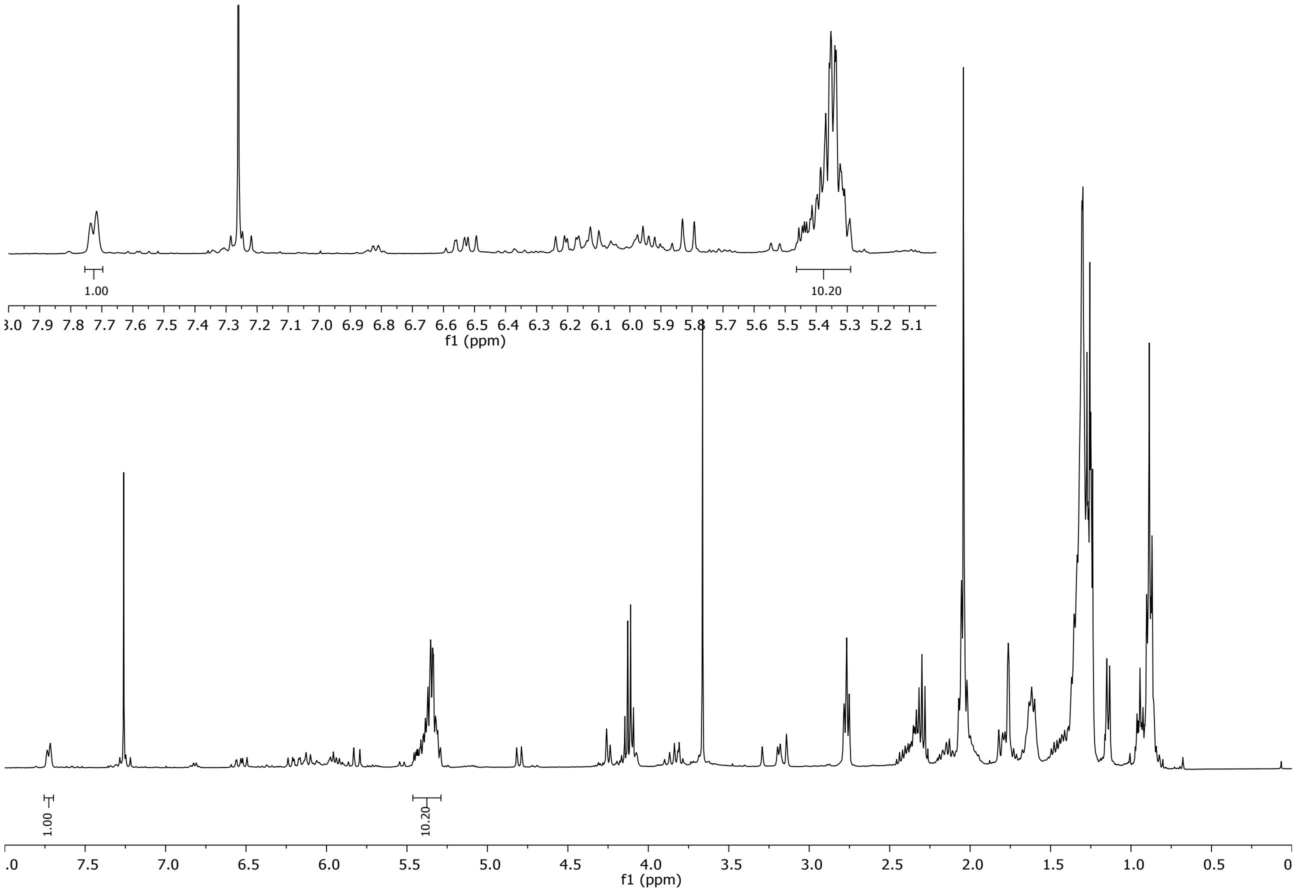

Figure S1.3 - ^{1}H NMR spectrum of the defatted *F. picrosperma* seed kernels extract by solid/liquid extraction

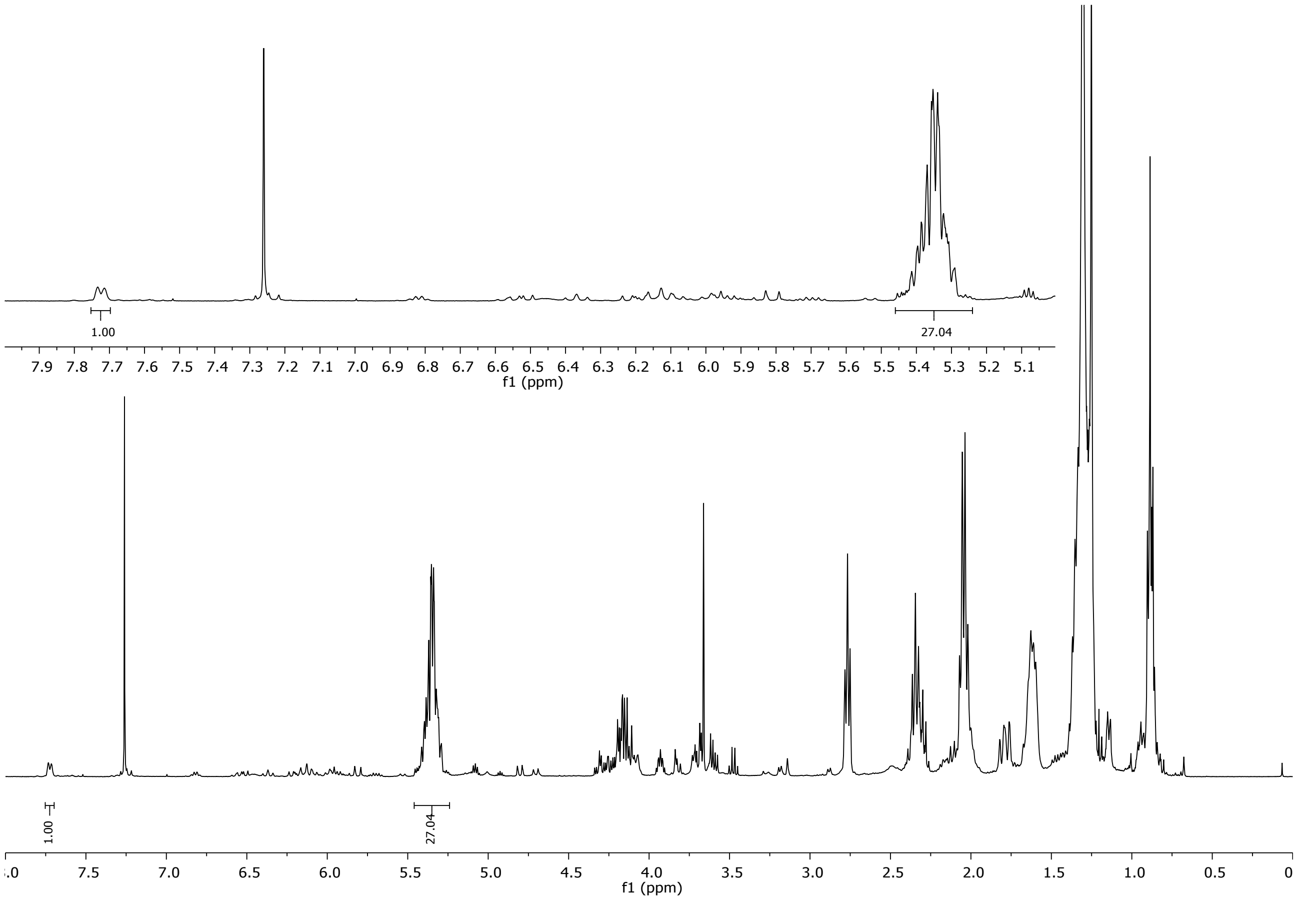

Figure S2 – a) (+)-HRESIMS spectra of **2a**, *m/z* 563.2852 $[M+H]^+$ b) (+)-HRESIMS/MS spectra of **2a**

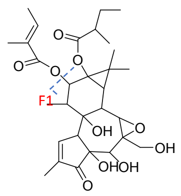

a)

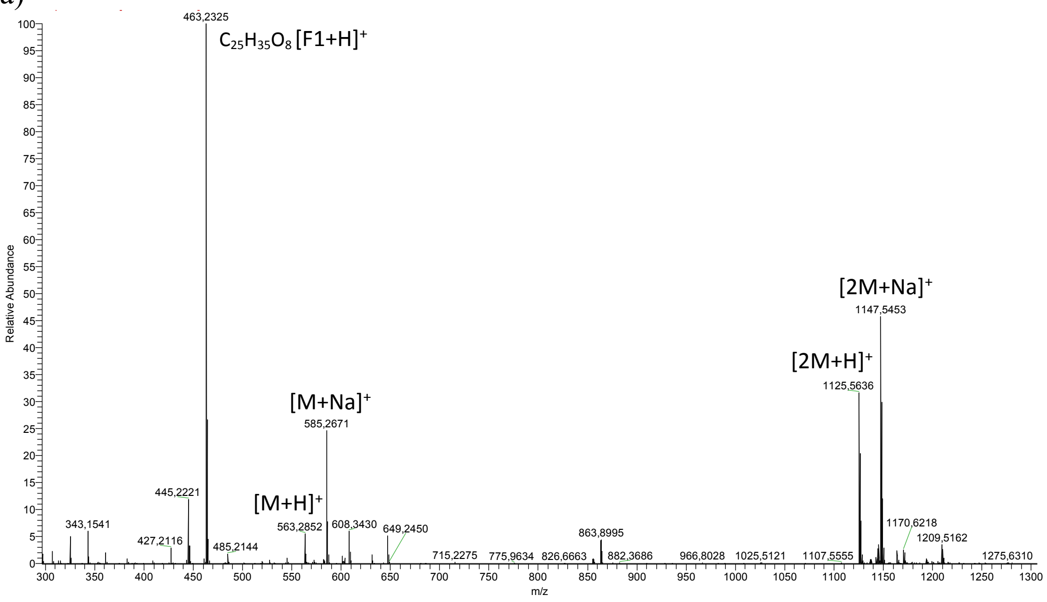

b)

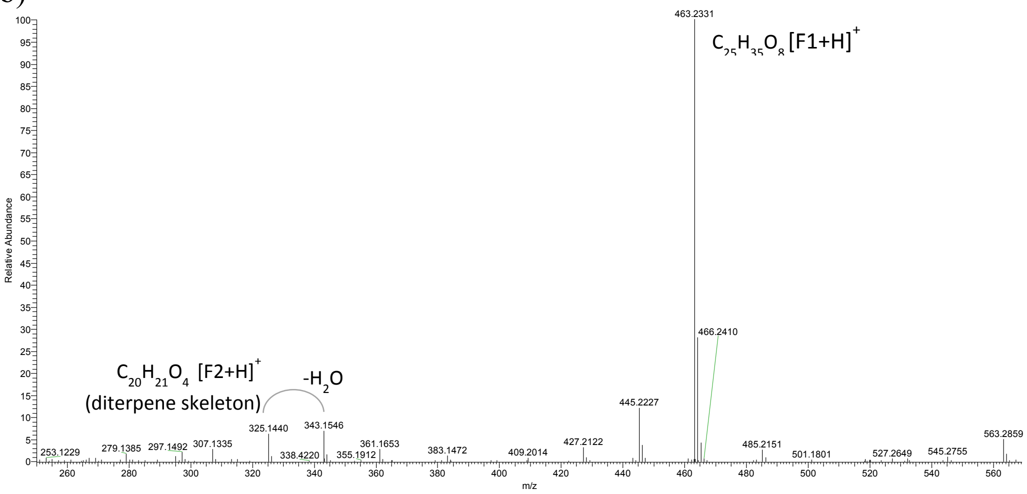

Figure S3 - a) (+)-HRESIMS spectra of **2b**, *m/z* 825.5157 $[M+H]^+$ b) (+)-HRESIMS/MS spectra of **2b**, *m/z* 825.51 $[M+H]^+$ (upper), and fragment F1, *m/z* 725.5 $[M+H]^+$ (lower)

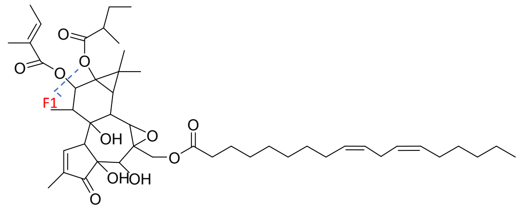

a)

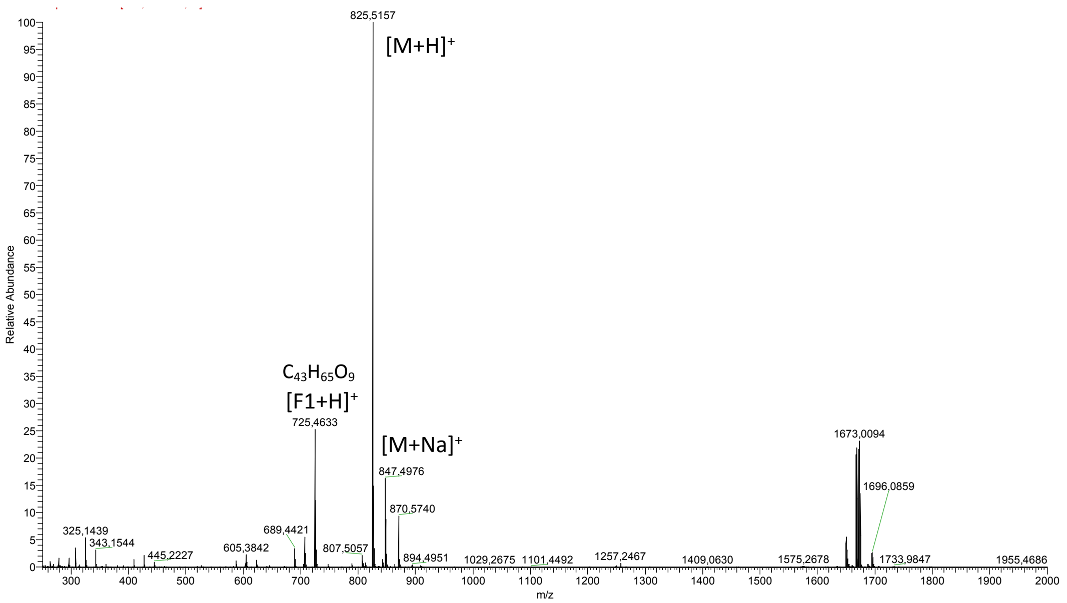

b)

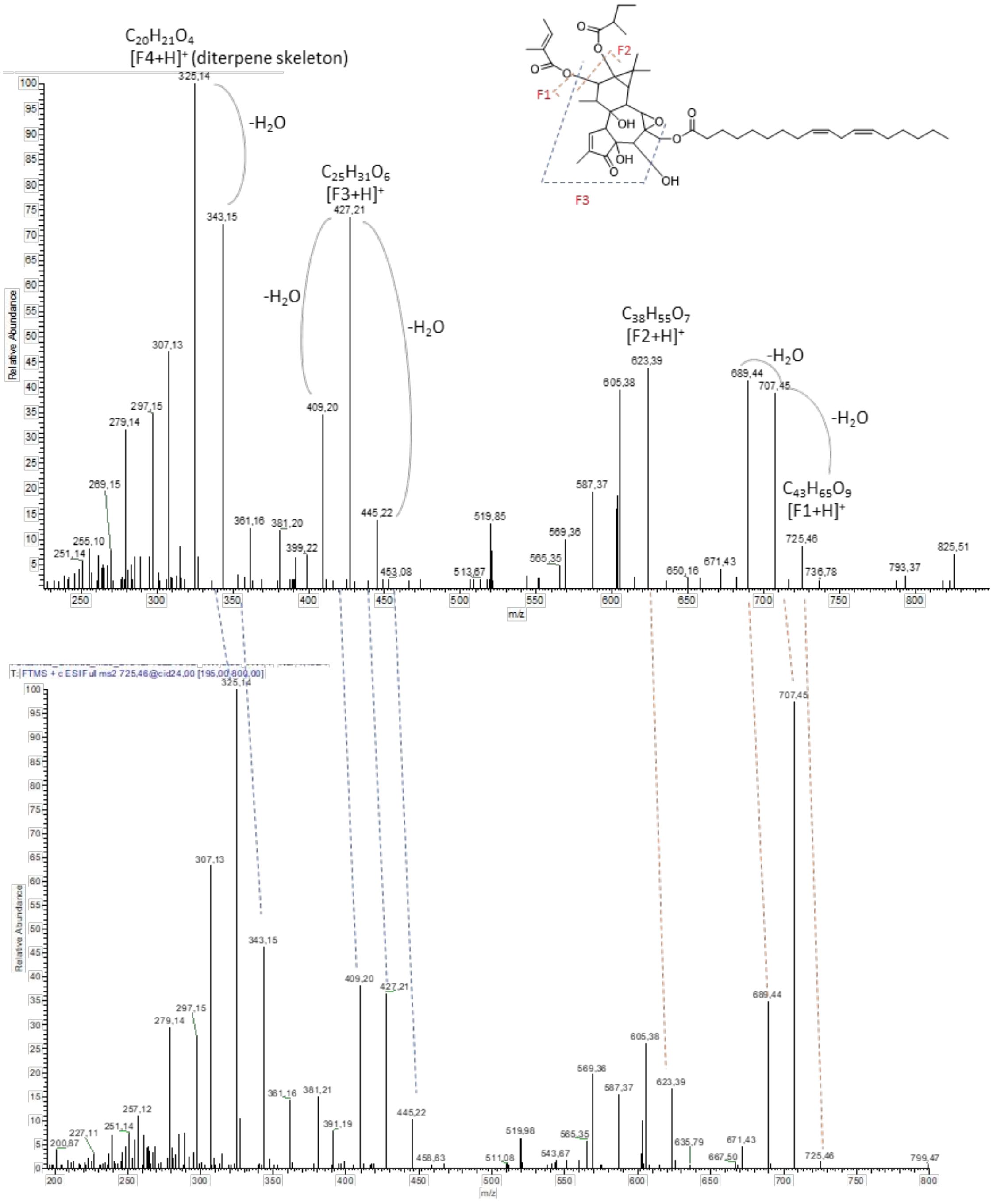

C20H21O4
[F4+H]+ (diterpene skeleton)
-H2O
C25H31O6
[F3+H]+
-H2O
-H2O
C38H55O7
[F2+H]+
-H2O
-H2O
C43H65O9
[F1+H]+
F1
F2
F3
Relative Abundance
m/z
T: FTMS + c ESI Full ms2 725,46@cid24,00 [195,00-800,00]

Figure S4 – a) (+)-HRESIMS spectra of **4a**, *m/z* 565.3016 $[M+H]^+$ b) (+)-HRESIMS/MS spectra of **4a**

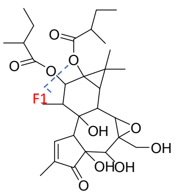

a)

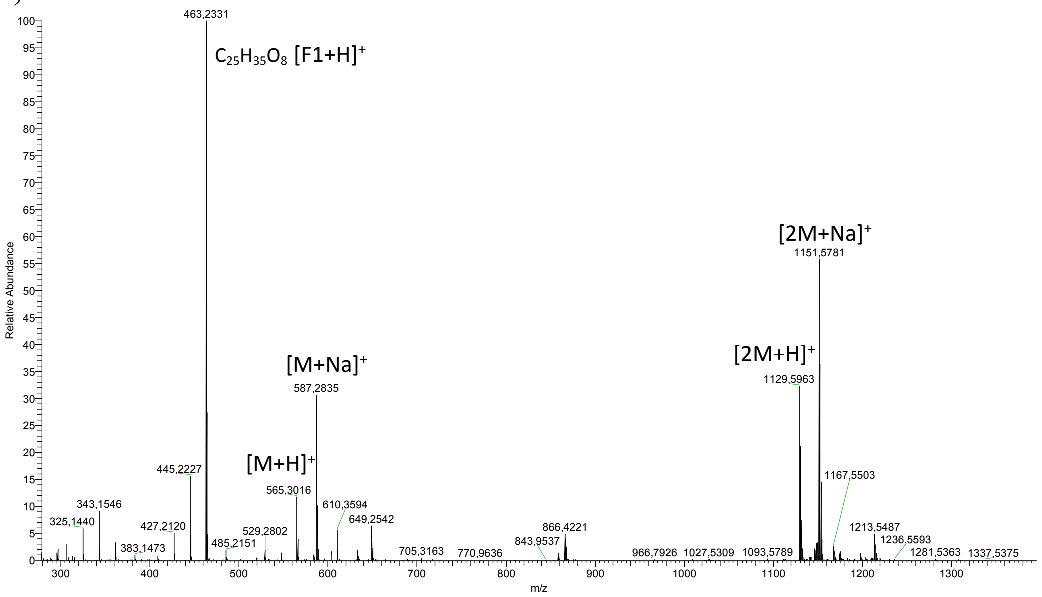

b)

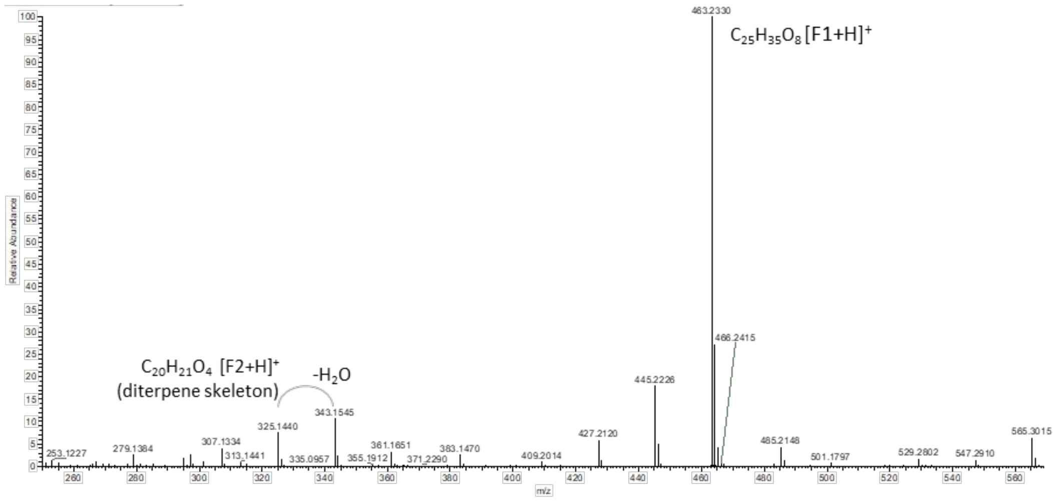

Figure S5 - a) (+)-HRESIMS spectra of **4b**, *m/z* 827.5327 [M+H]+ b) (+)-HRESIMS/MS spectra of **4b**, *m/z* 827.53 [M+H]+ (upper), and fragment F1, *m/z* 725.5 [M+H]+ (lower)

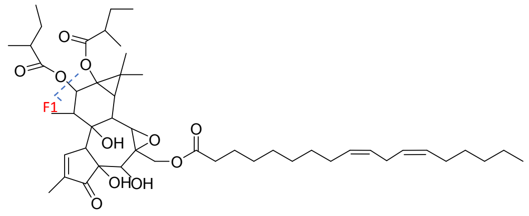

a)

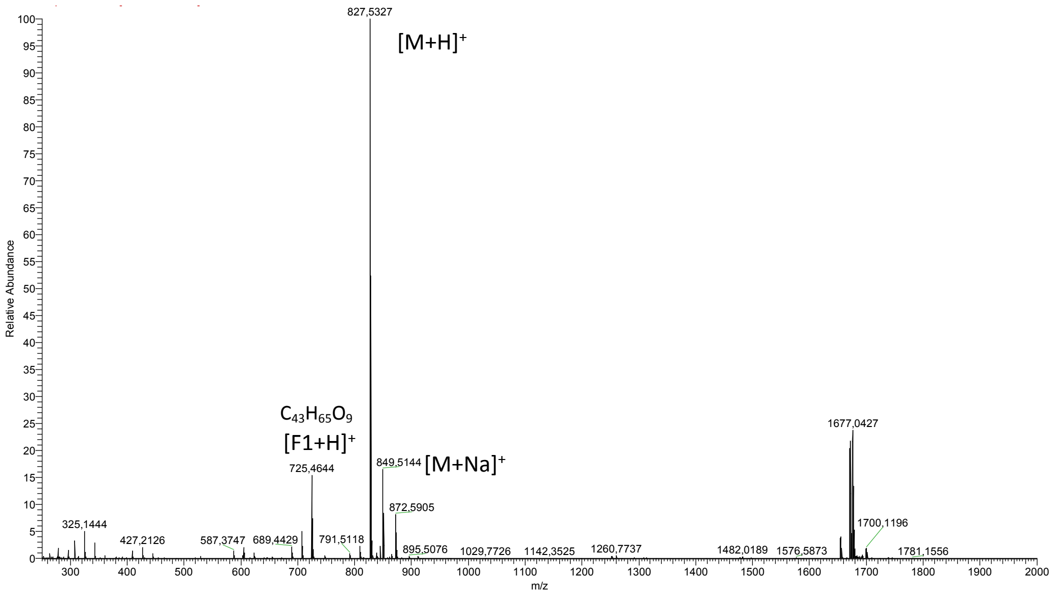

b)

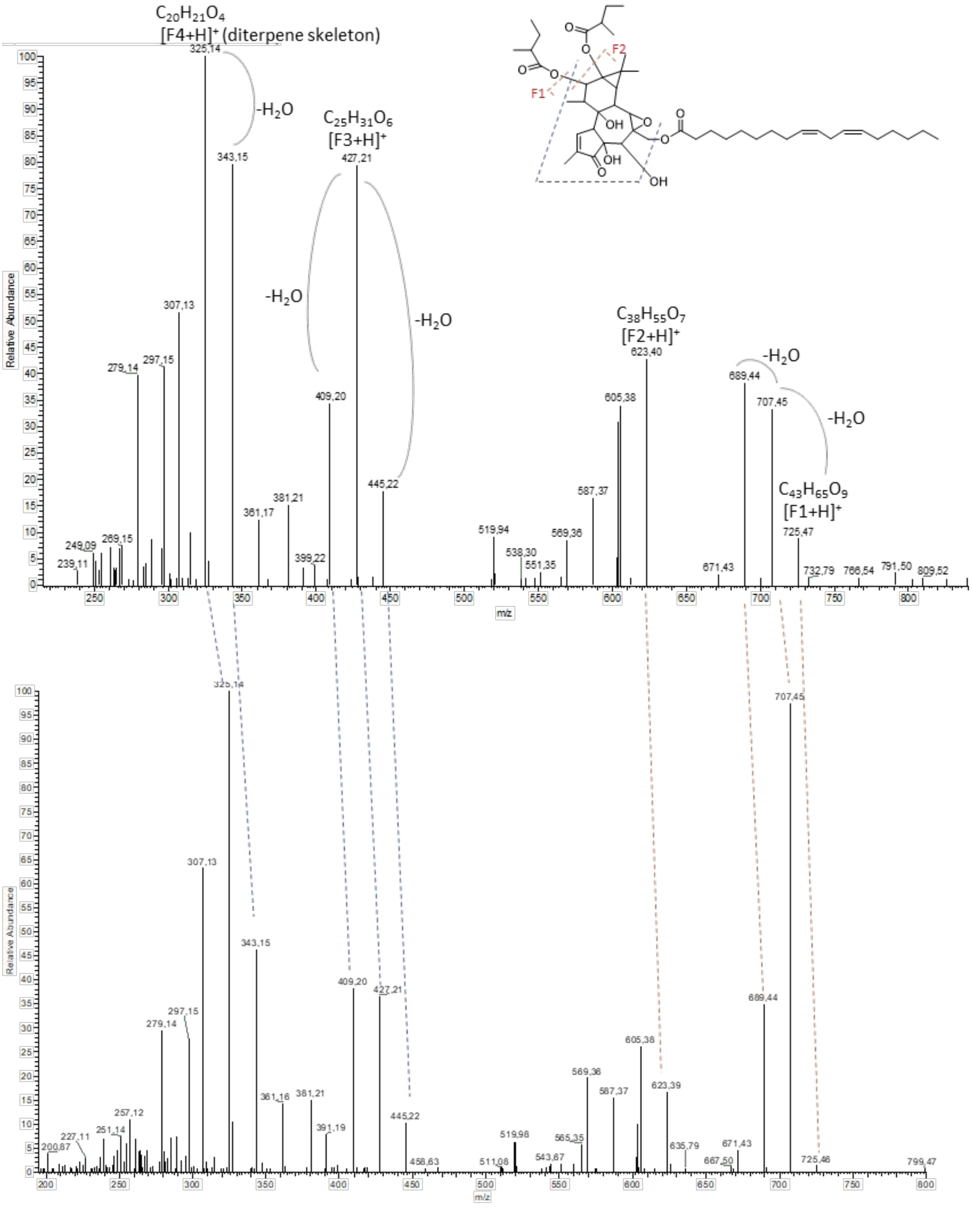

C20H21O4
[F4+H]+ (diterpene skeleton)
C25H31O6
[F3+H]+
C38H55O7
[F2+H]+
C43H65O9
[F1+H]+
-H2O
-H2O
-H2O
-H2O
-H2O
F1
F2
OH
OH
OH
O
Relative Abundance
m/z
325,14
343,15
427,21
409,20
445,22
307,13
297,15
279,14
381,21
361,17
399,22
269,15
249,09
239,11
519,94
538,30
551,35
569,36
587,37
605,38
623,40
671,43
689,44
707,45
725,47
732,79
766,54
791,50
809,52
361,16
391,19
257,12
251,14
227,11
200,87
458,63
511,08
519,98
543,67
565,35
623,39
635,79
667,50
725,46
799,47

Figure S6 - a) (+)-HRESIMS spectra of **4c**, *m/z* 829.5470 $[M+H]^+$ b) (+)-HRESIMS/MS spectra of **4c**, *m/z* 829.55 $[M+H]^+$ (upper), and fragment F1, *m/z* 727.5 $[M+H]^+$ (lower)

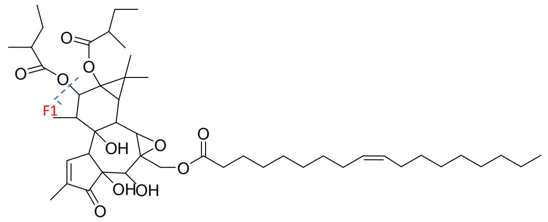

a)

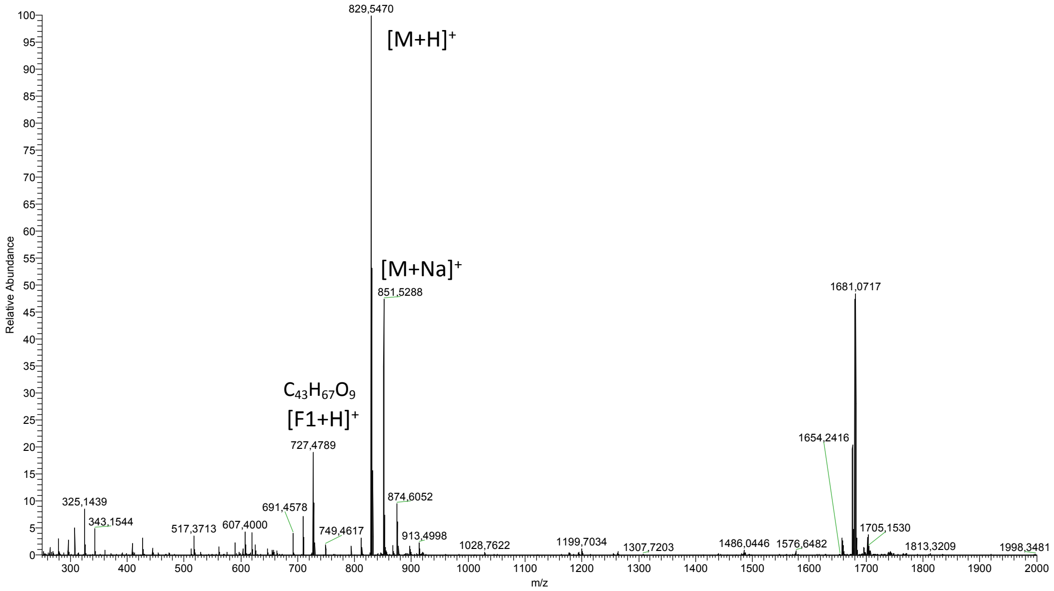

b)

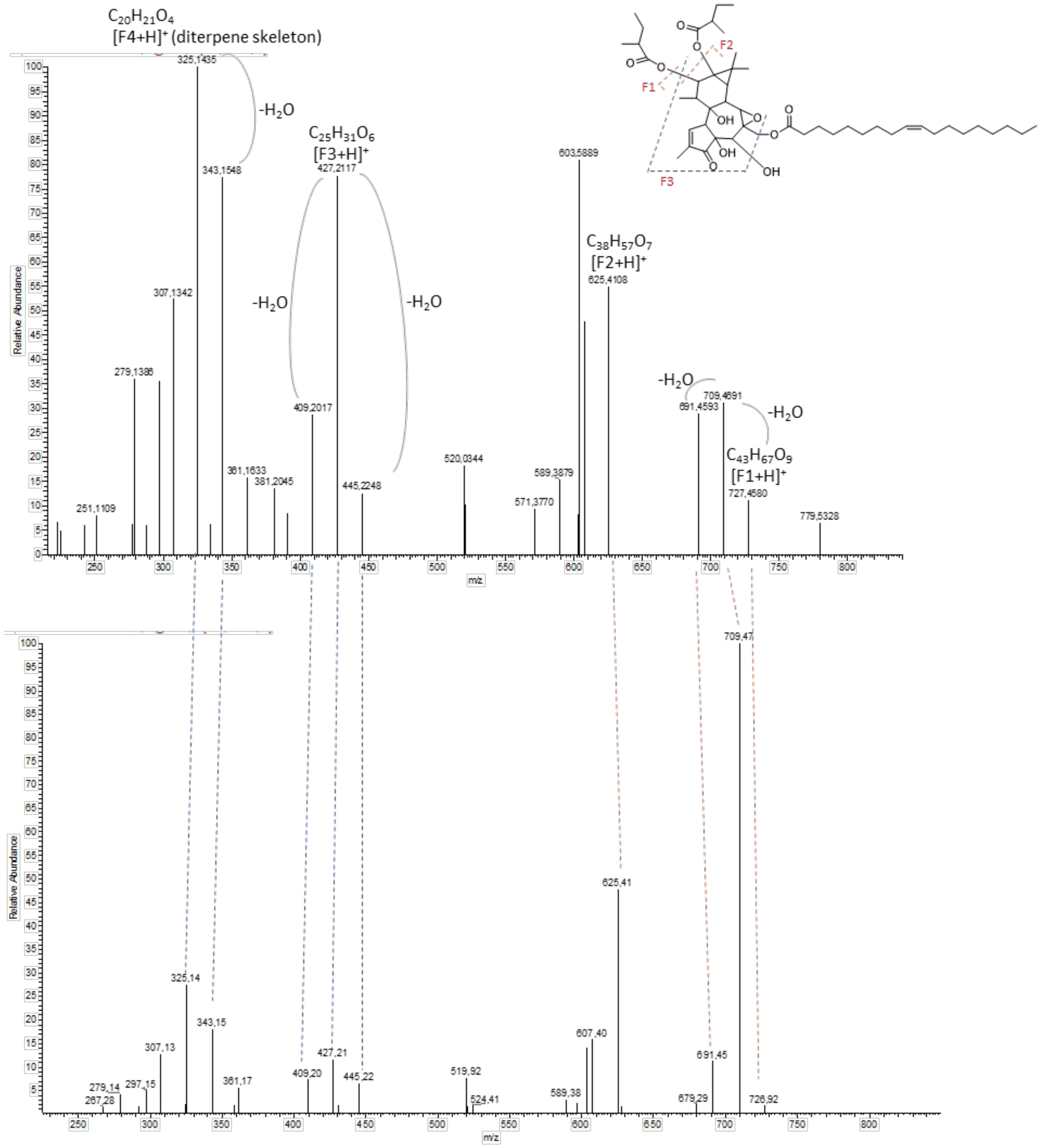

C20H21O4
[F4+H]+ (diterpene skeleton)
-H2O
C25H31O6
[F3+H]+
C38H57O7
[F2+H]+
C43H67O9
[F1+H]+
F1
F2
F3
OH
325,1435
343,1548
307,1342
279,1386
251,1109
361,1633
381,2045
409,2017
427,2117
445,2248
520,0344
571,3770
589,3879
603,5889
625,4108
691,4593
709,4691
727,4580
779,5328
Relative Abundance
m/z
709,47
625,41
325,14
343,15
307,13
297,15
279,14
267,28
361,17
409,20
427,21
445,22
519,92
524,41
589,38
607,40
679,29
691,45
726,92

Figure S7 – a) (+)-HRESIMS spectra of **5a**, *m/z* 657.3640 $[M+H]^+$ b) (+)-HRESIMS/MS spectra of **5a**

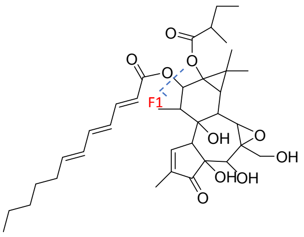

a)

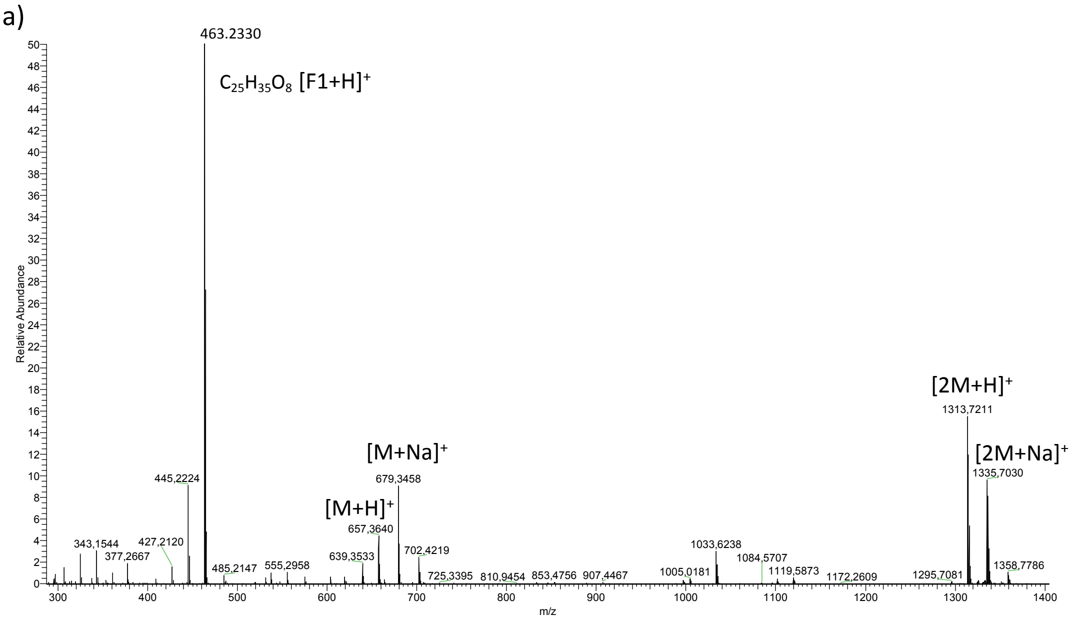

b)

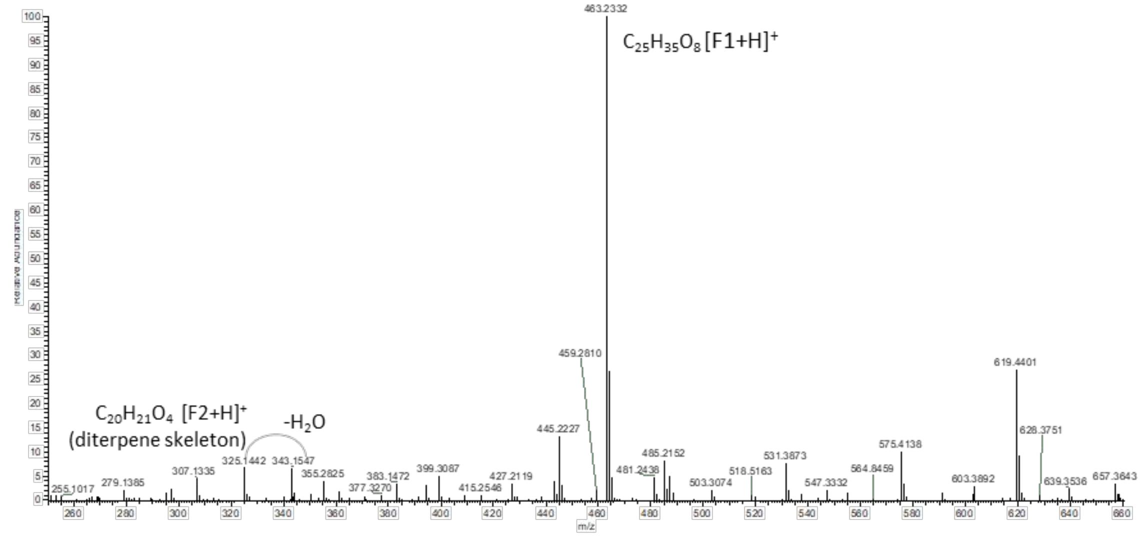

Figure S8 – a) (+)-HRESIMS spectra of **5b**, *m/z* 919.5944 $[M+H]^+$ b) (+)-HRESIMS/MS spectra of **5b**

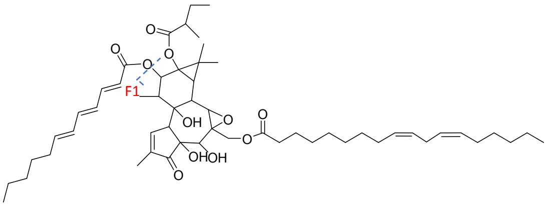

a)

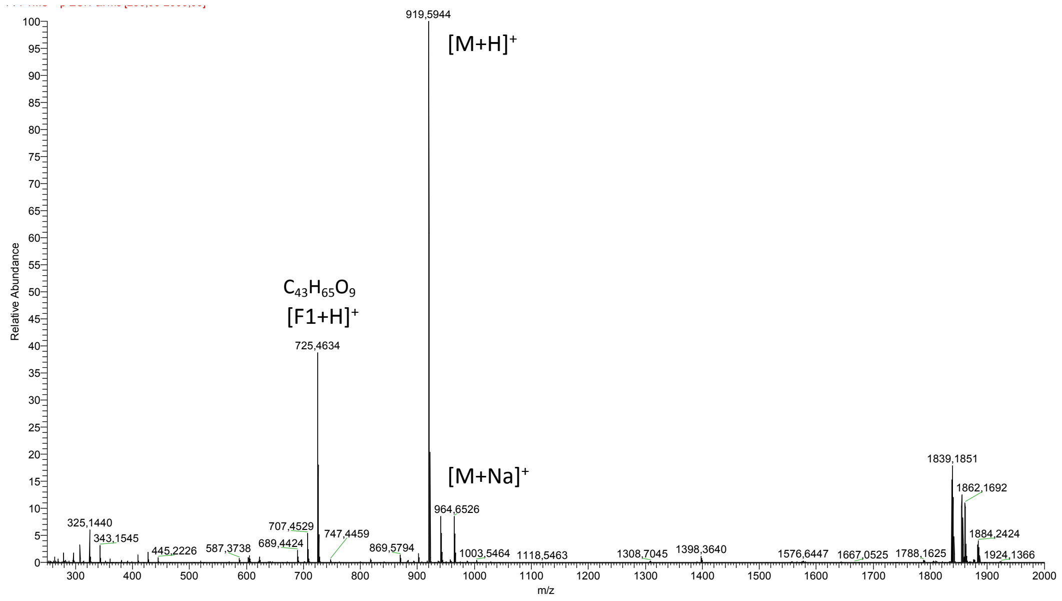

b)

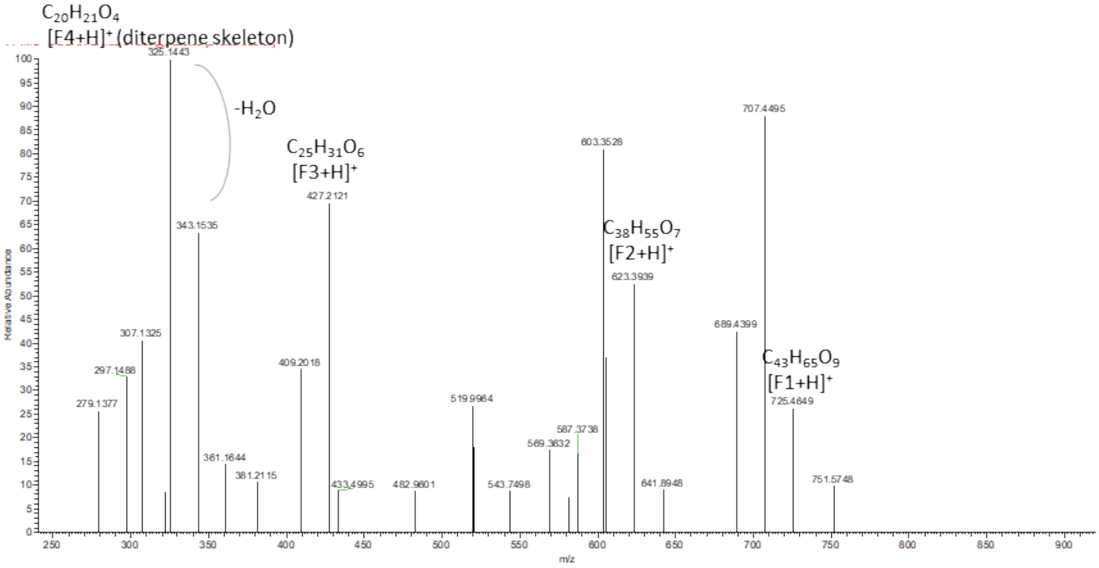

Figure S9 – a) (+)-HRESIMS spectra of **5c**, *m/z* 921.6097 $[M+H]^+$ b) (+)-HRESIMS/MS spectra of **5c**

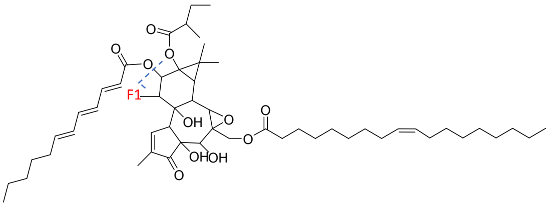

a)

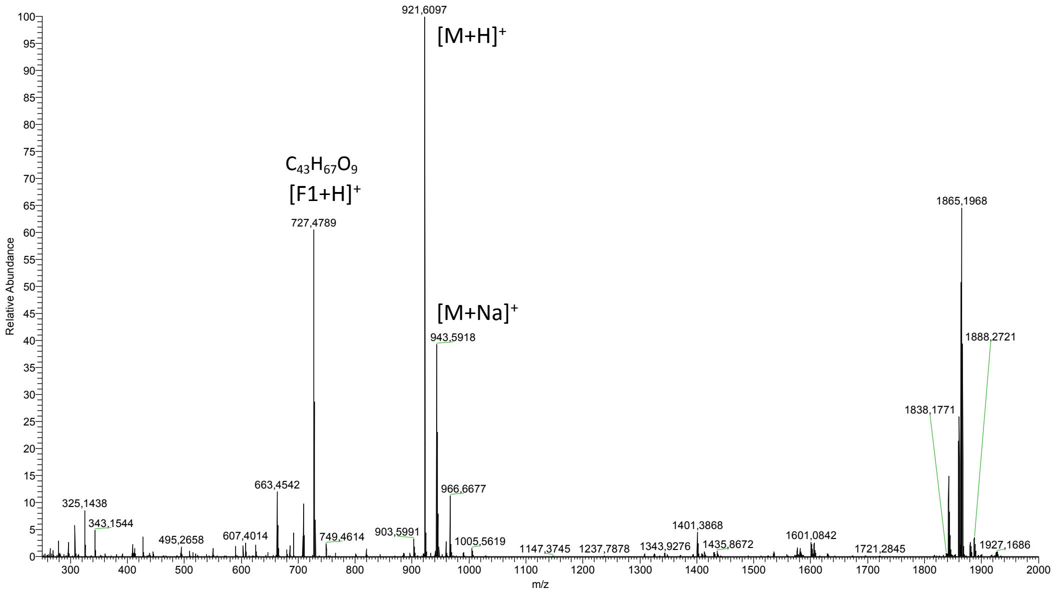

b)

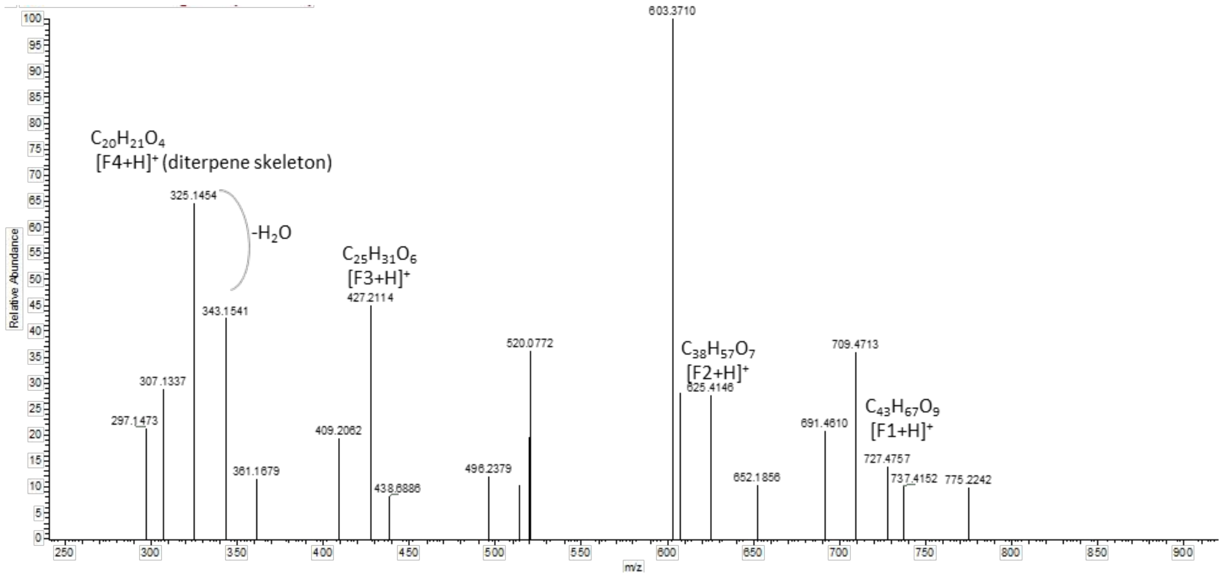

Figure S10 – a) (+)-HRESIMS spectra of **6a**, *m/z* 579.3173 $[M+H]^+$ b) (+)-HRESIMS/MS spectra of **6a**

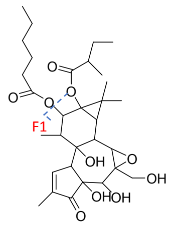

a)

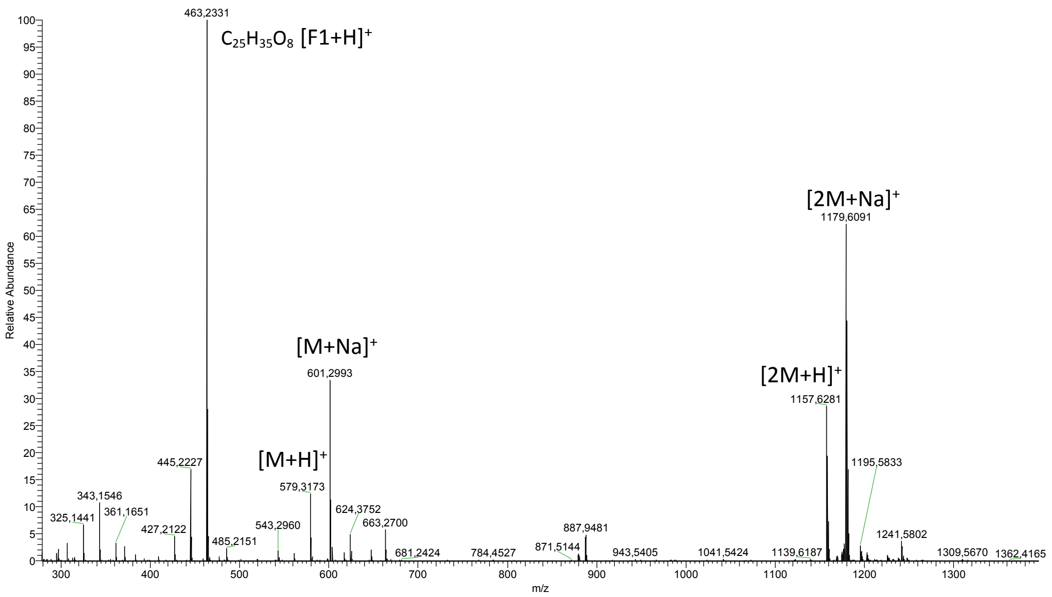

b)

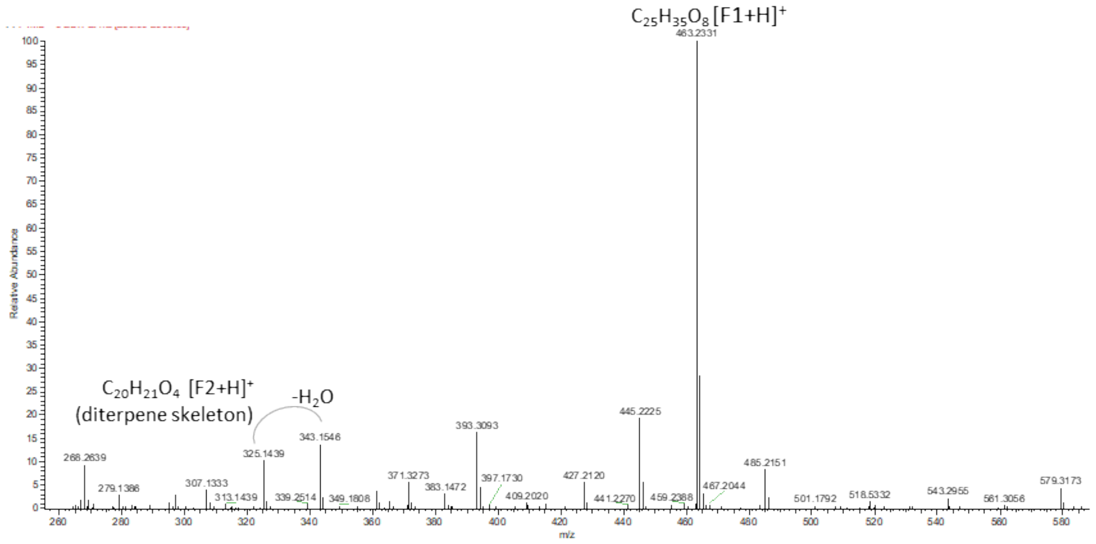

Figure S11 – a) (+)-HRESIMS spectra of **6b**, *m/z* 841.5474 $[M+H]^+$ b) (+)-HRESIMS/MS spectra of **6b**

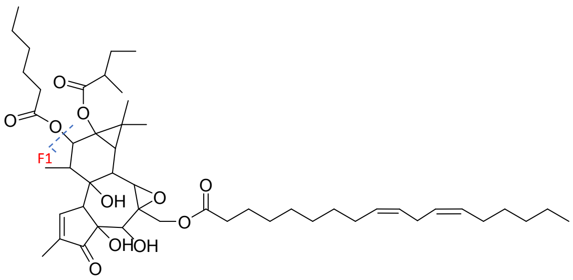

a)

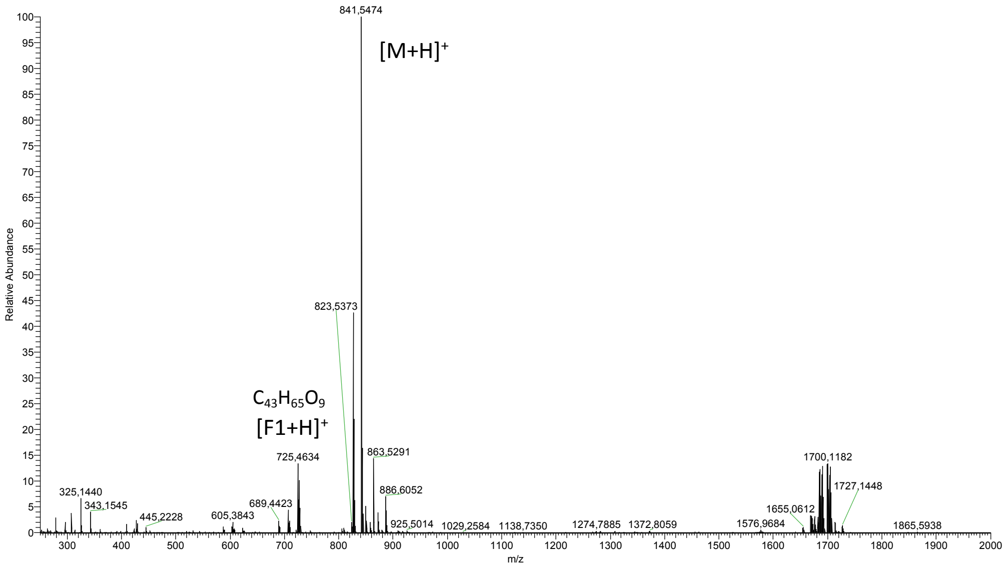

b)

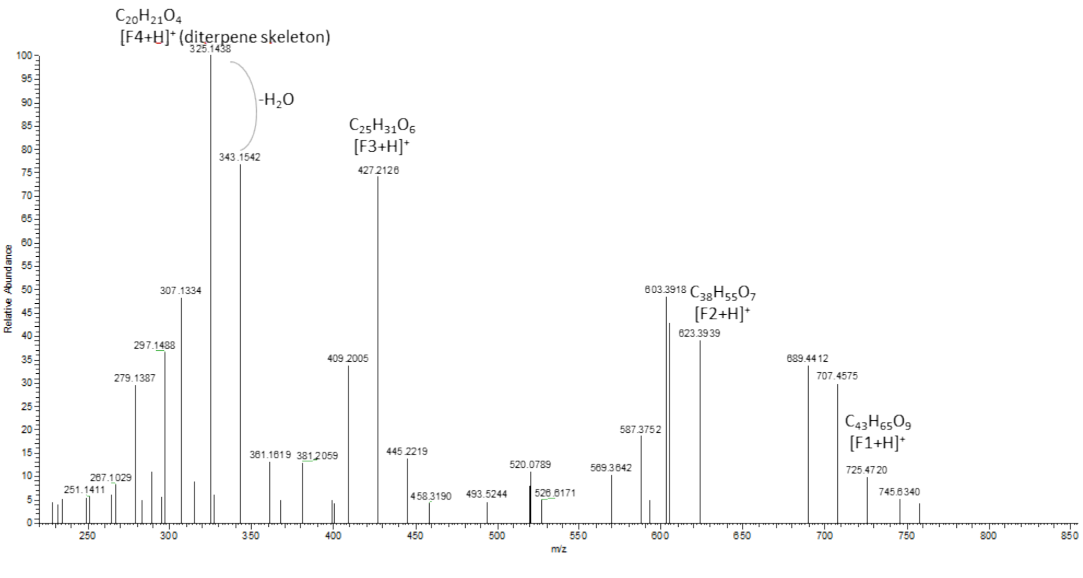

Figure S12 – a) (+)-HRESIMS spectra of **6c**, *m/z* 843.5634 [M+H]$^+$ b) (+)-HRESIMS/MS spectra of **6c**

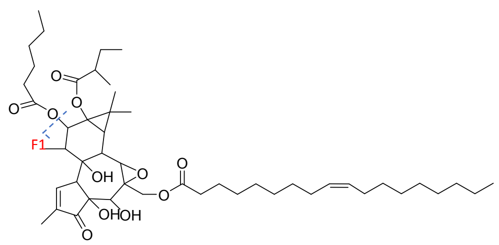

a)

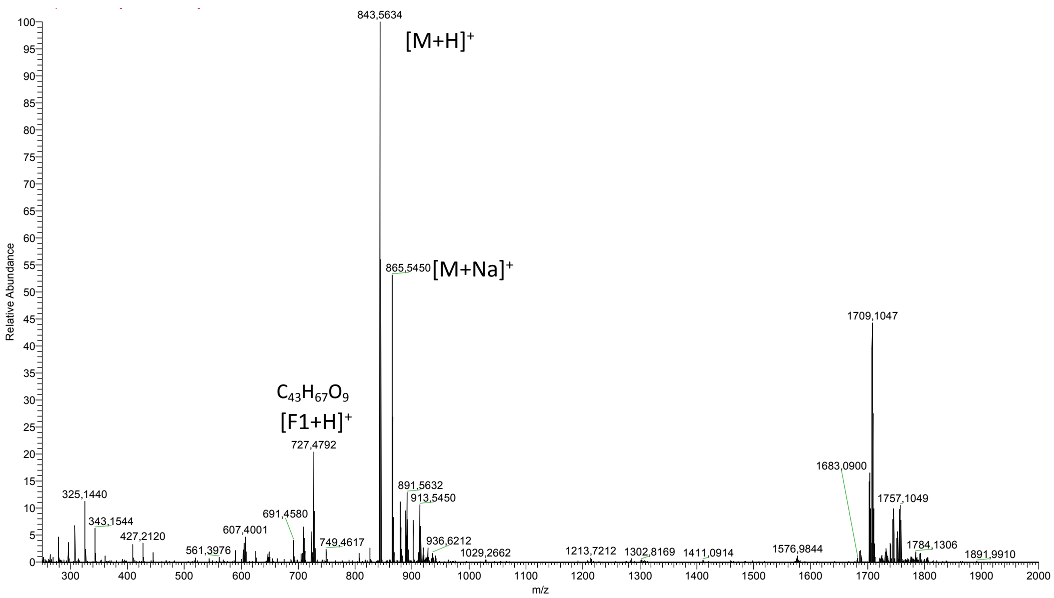

b)

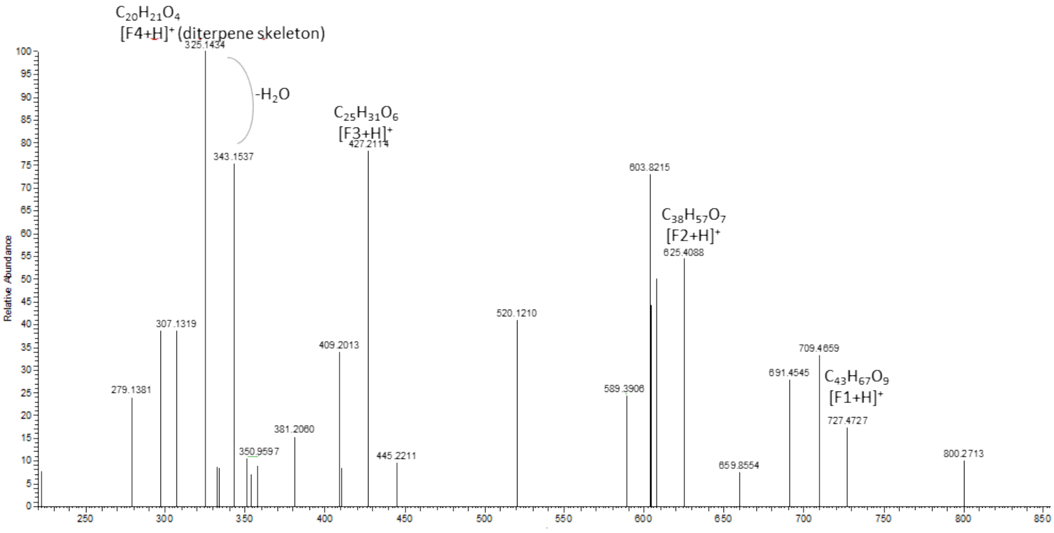

Figure S13 – a) (+)-HRESIMS spectra of **7a**, *m/z* 631.3486 $[M+H]^+$ b) (+)-HRESIMS/MS spectra of **7a**

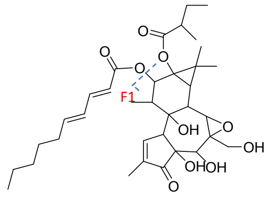

a)

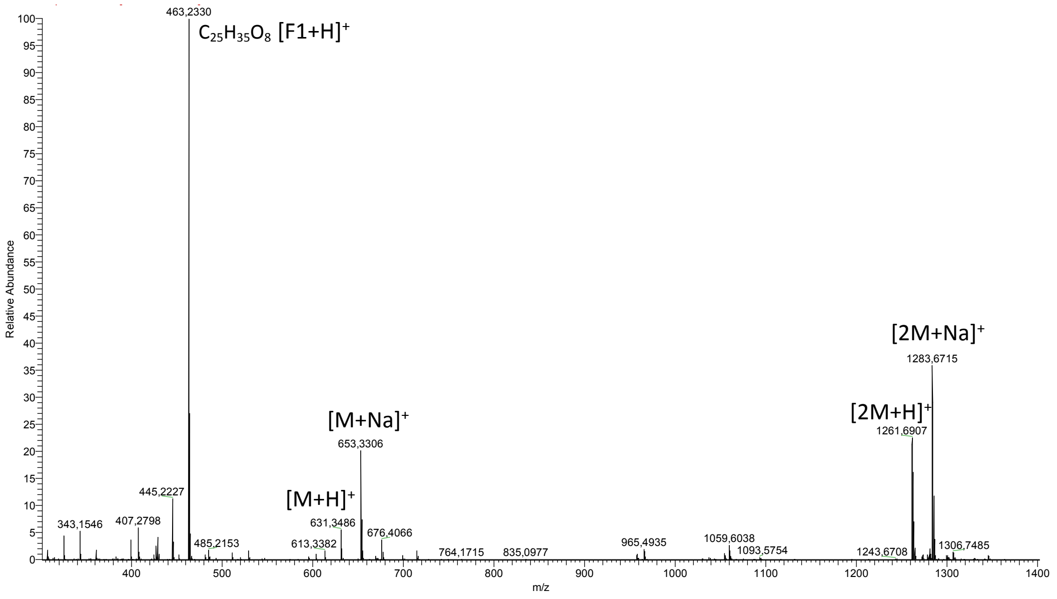

b)

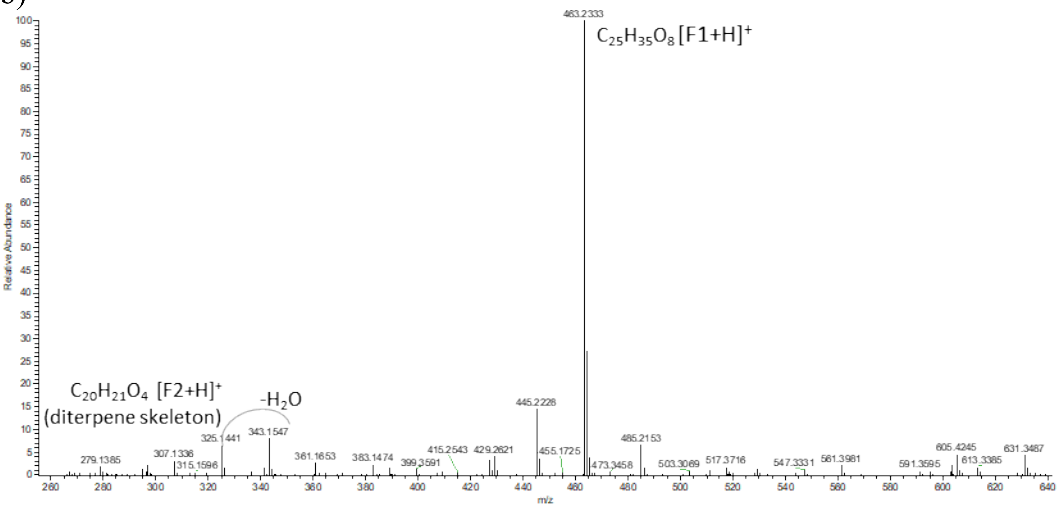

Figure S14 – a) (+)-HRESIMS spectra of **7b**, *m/z* 893.5786 $[M+H]^+$ b) (+)-HRESIMS/MS spectra of **7b**

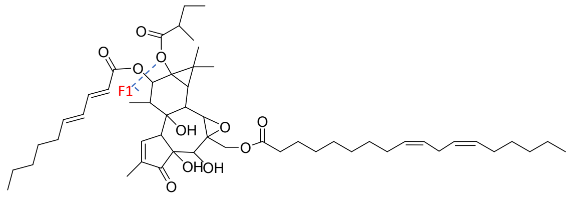

a)

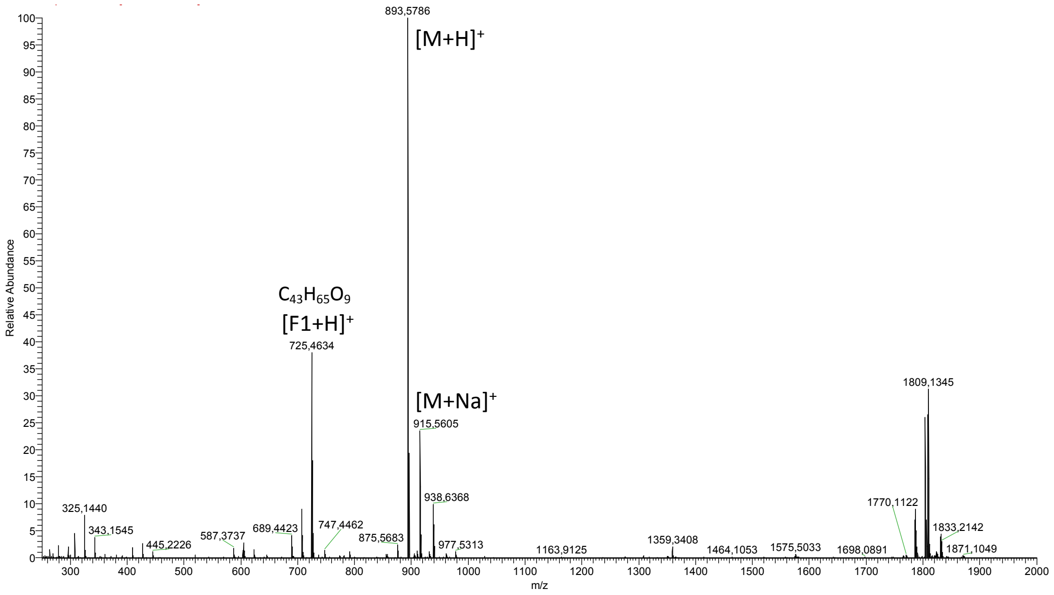

b)

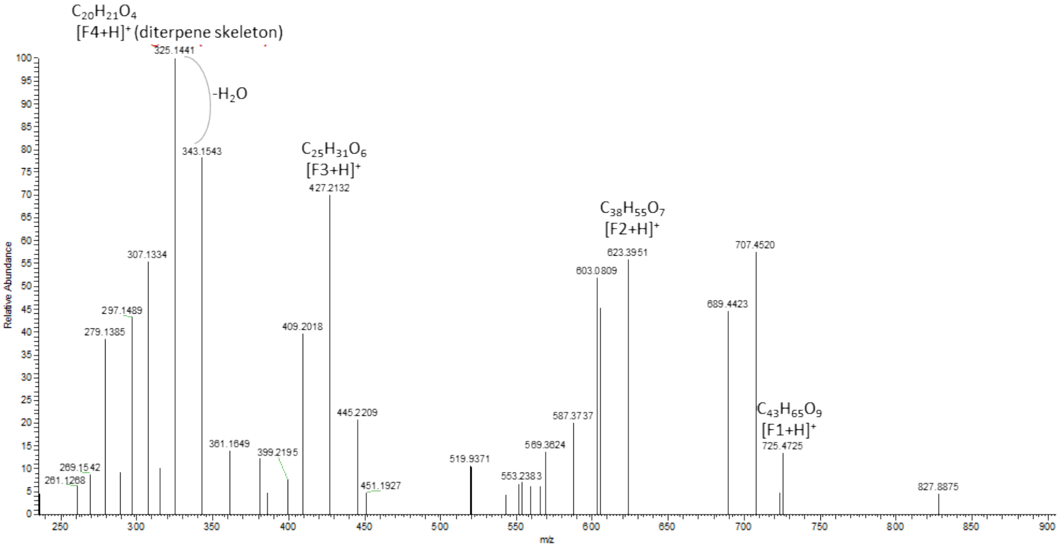

Figure S15 – a) (+)-HRESIMS spectra of **7c**, *m/z* 895.5955 $[M+H]^+$ b) b) (+)-HRESIMS/MS spectra of **7c**

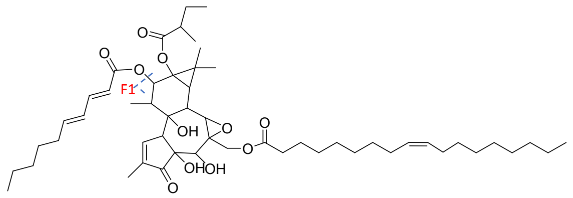

a)

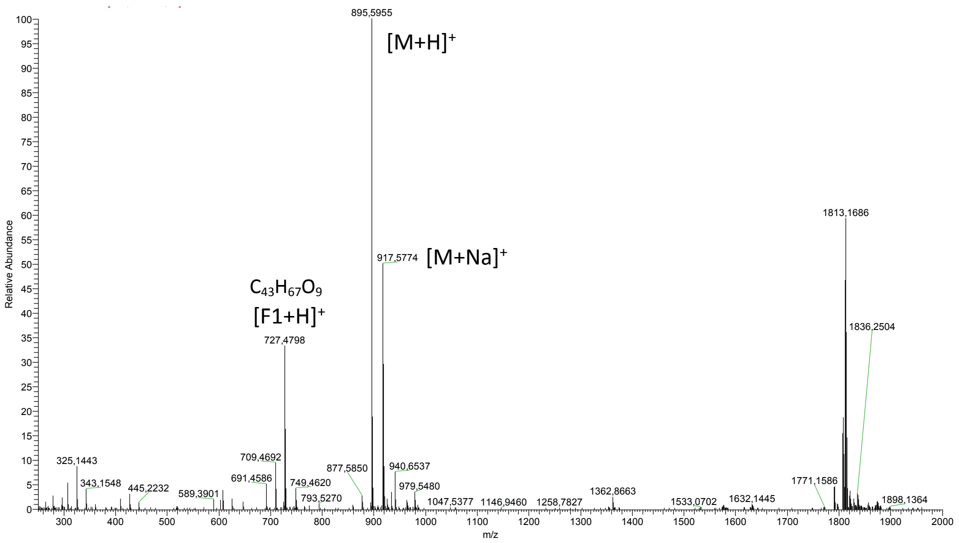

b)

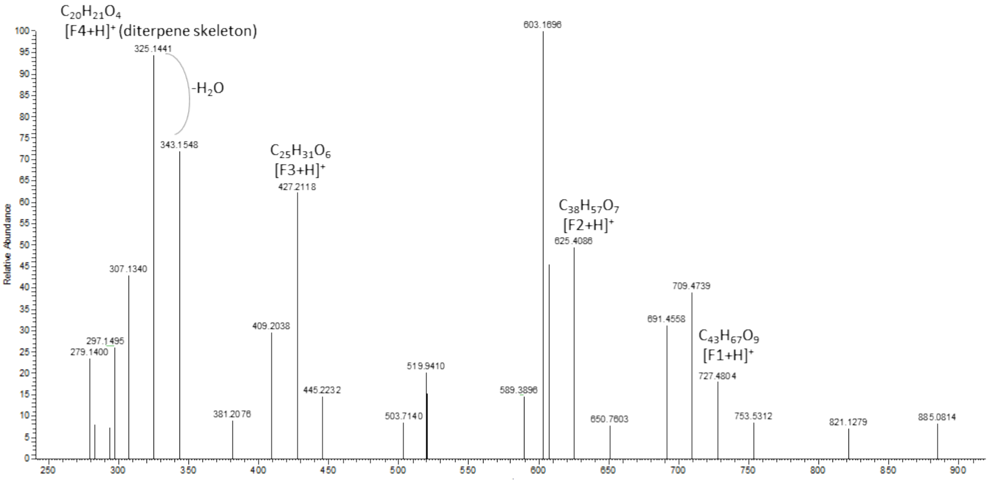

Figure S16 – Semisynthetic reaction starting from EBC-46 (2a) – Steglich reaction

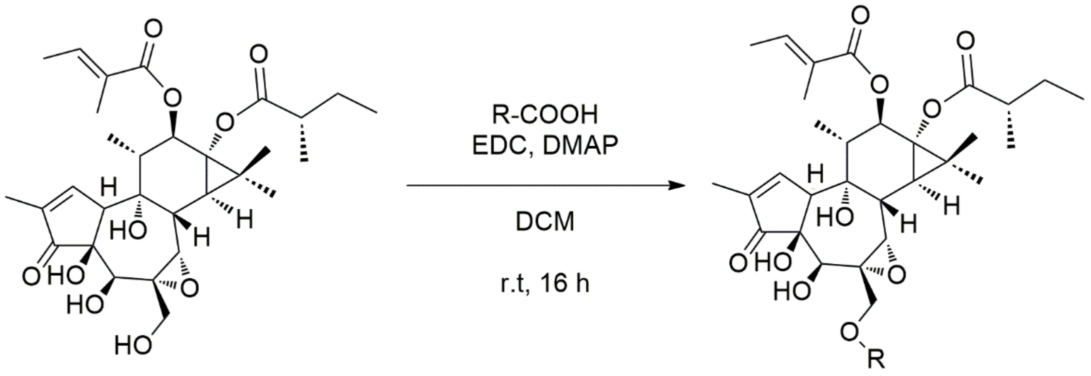

Entry	Residue	Yield
**2b**	Linoleic	67%
**2c**	Oleic	46%
**2d**	Linolenic	37%
**2e**	Palmitic	31%
**2f**	Myristic	79%

Figure S17 - Semisynthetic reaction starting from EBC-59 (**5a**) – Steglich reaction

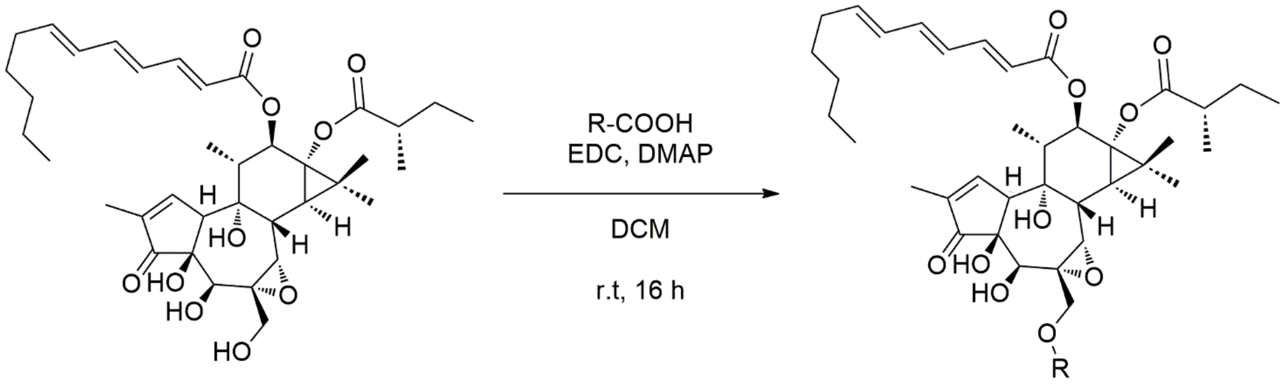

Entry	Residue	Yield
**5b**	Linoleic	57%
**5c**	Oleic	63%
**5d**	Linolenic	59%
**5e**	Stearic	30%

Figure S18 - ^{1}H NMR spectrum of 12-tigloyl-13-(2-methylbutyryl)-5β-hydroxy-6α,7α -epoxyphorbol, EBC-46, **2a** (400 MHz, $CDCl_3$)

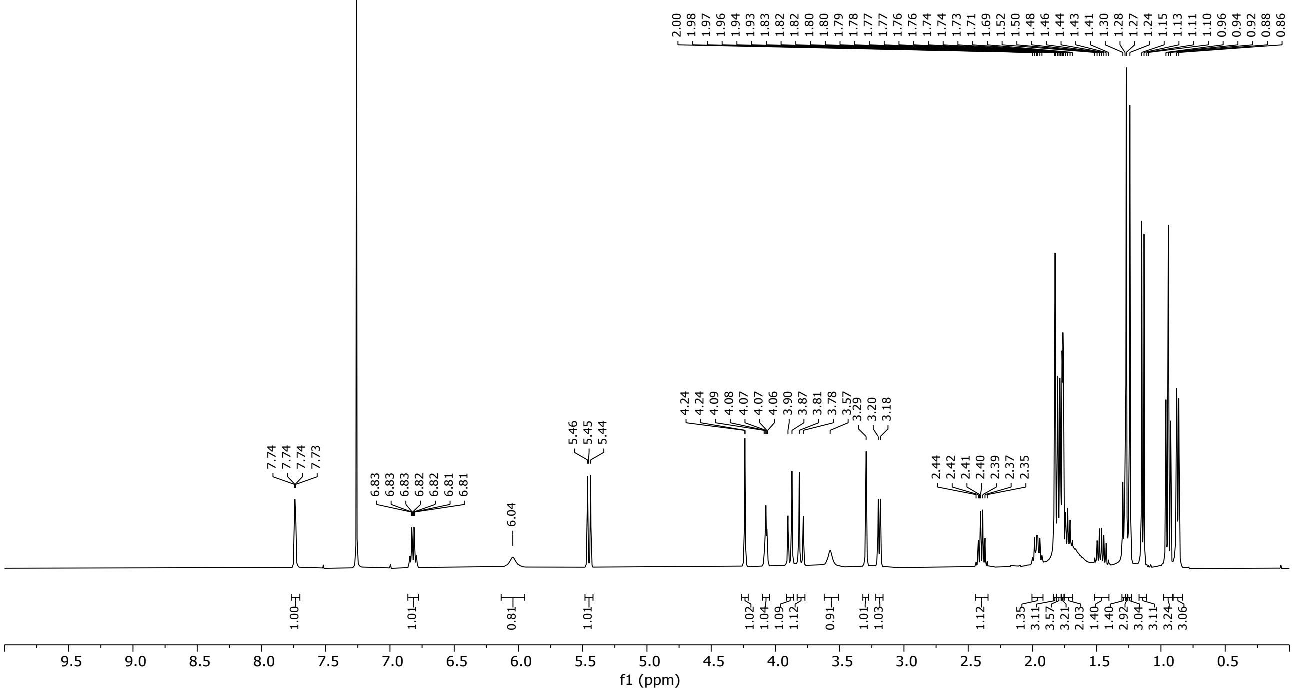

Figure S18.1 - ^{1}H NMR spectrum of 12-tigloyl-13-(2-methylbutyryl)-5β-hydroxy-6α,7α -epoxyphorbol-20-oleate, **2c** (400 MHz, $CDCl_3$)

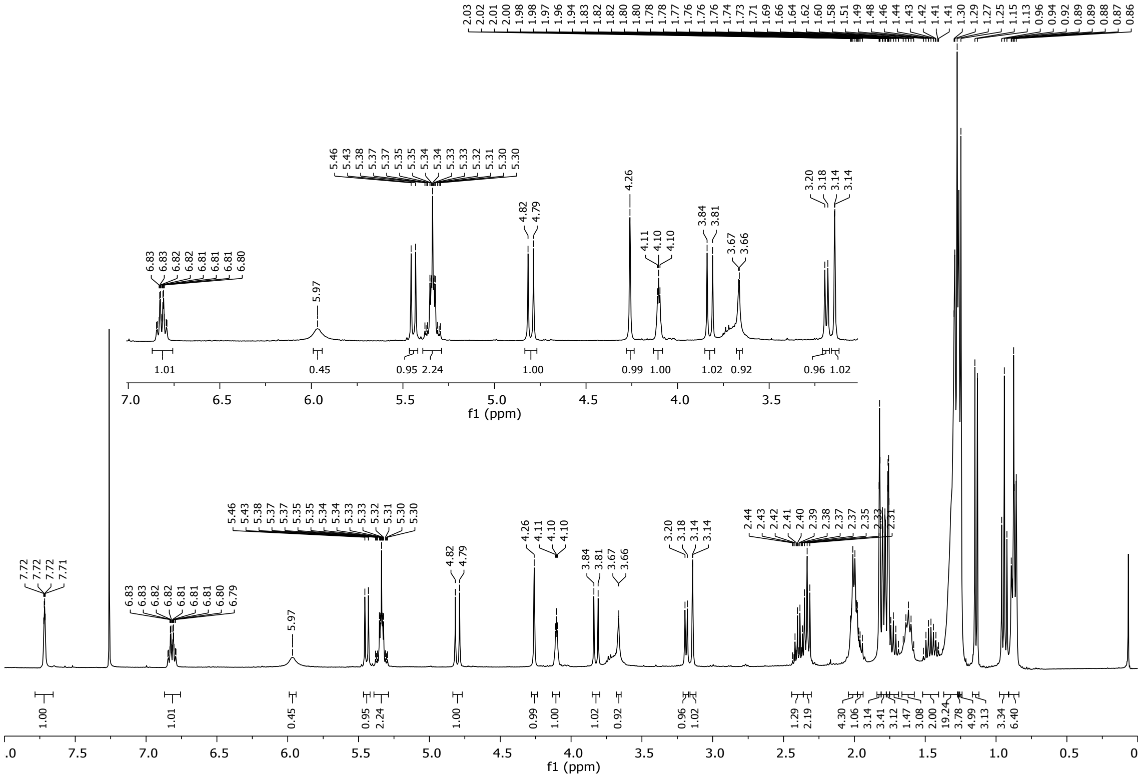

Figure S18.2 – ^{13}C NMR spectrum of 12-tigloyl-13-(2-methylbutyryl)-5β-hydroxy-6α,7α -epoxyphorbol-20-oleate, **2c** (125 MHz, $CDCl_3$)

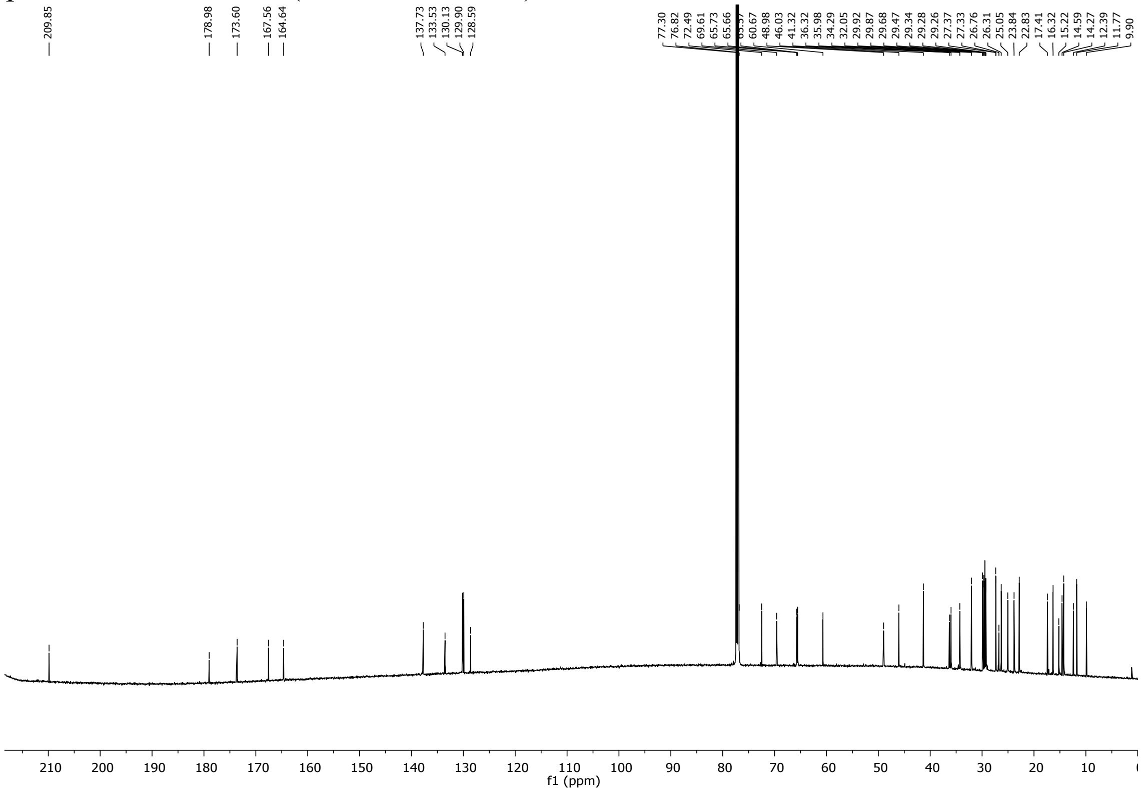

Figure S19.1 - ^{1}H NMR spectrum of 12-tigloyl-13-(2-methylbutyryl)-5β-hydroxy-6α,7α - epoxyphorbol-20-linolenate, **2d** (400 MHz, $CDCl_3$)

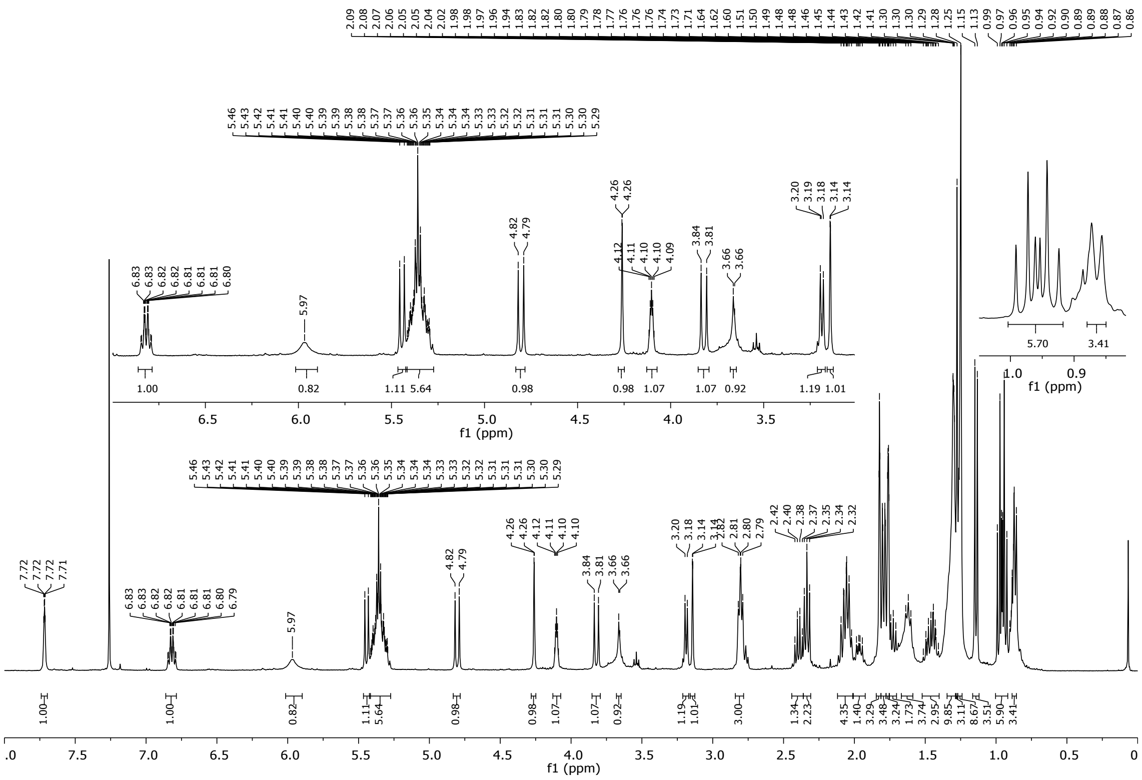

Figure S19.2 - ^{13}C NMR spectrum of 12-tigloyl-13-(2-methylbutyryl)-5β-hydroxy-6α,7α - epoxyphorbol-20-linolenate, **2d** (125 MHz, $CDCl_3$)

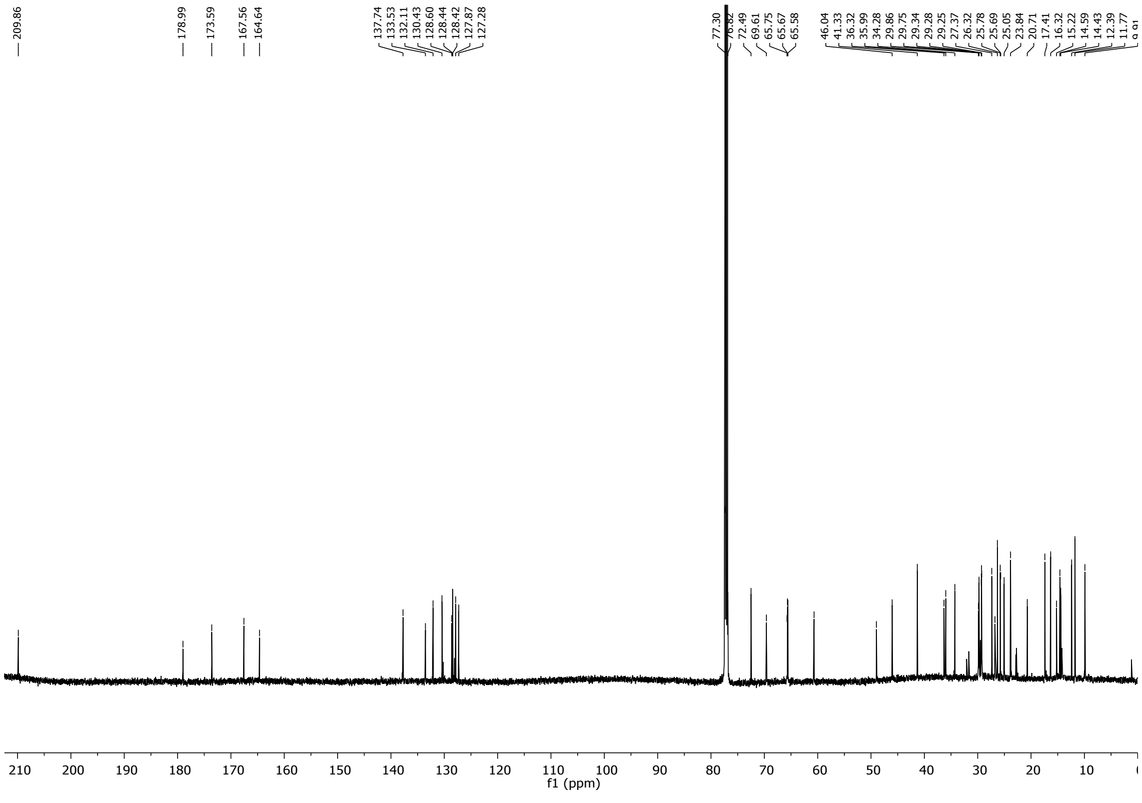

Figure S20.1 – ^{1}H NMR spectrum of 12-(2*E*,4*E*,6*E*)-dodecatrienoyl-13-(2-methylbutyryl)-5β-hydroxy-6α,7α-epoxyphorbol -20-stearate, **5e** (400 MHz, $CDCl_3$).

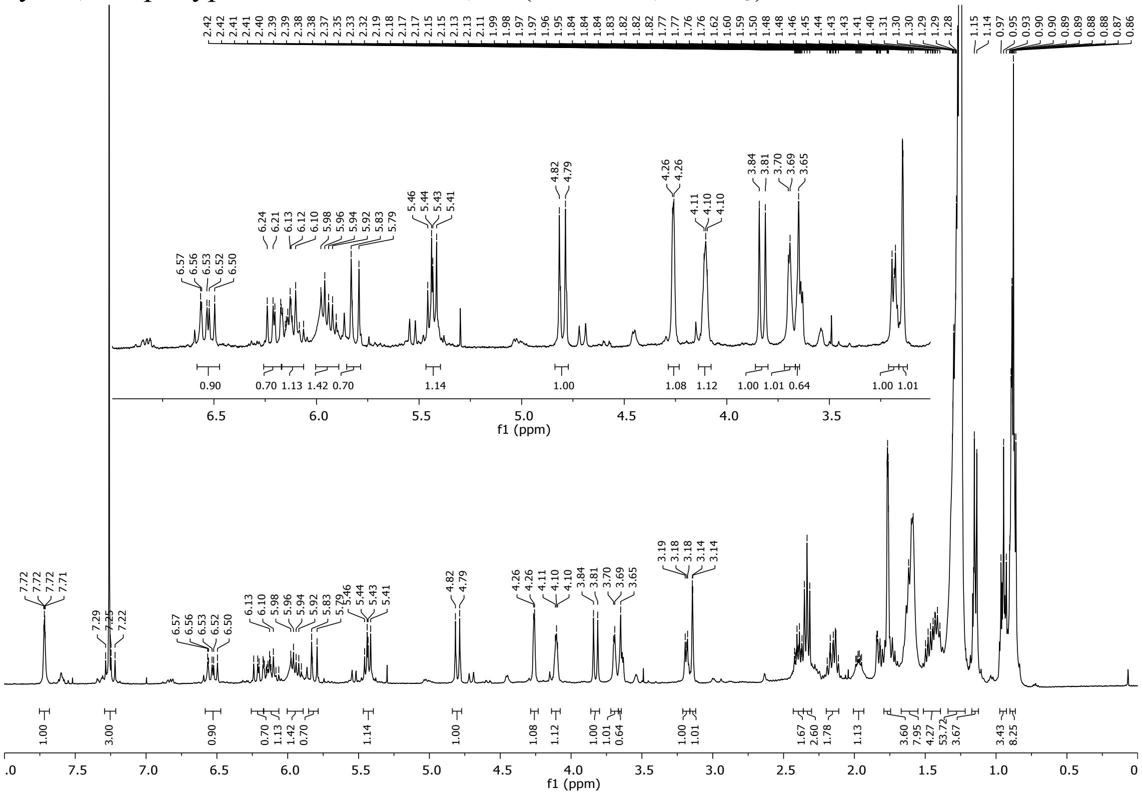

Figure S20.2 – ^{1}H NMR spectrum of 12-(2*E*,4*E*,6*E*)-dodecatrienoyl-13-(2-methylbutyryl)-5β-hydroxy-6α,7α-epoxyphorbol -20-stearate, **5e** (100 MHz, $CDCl_3$).

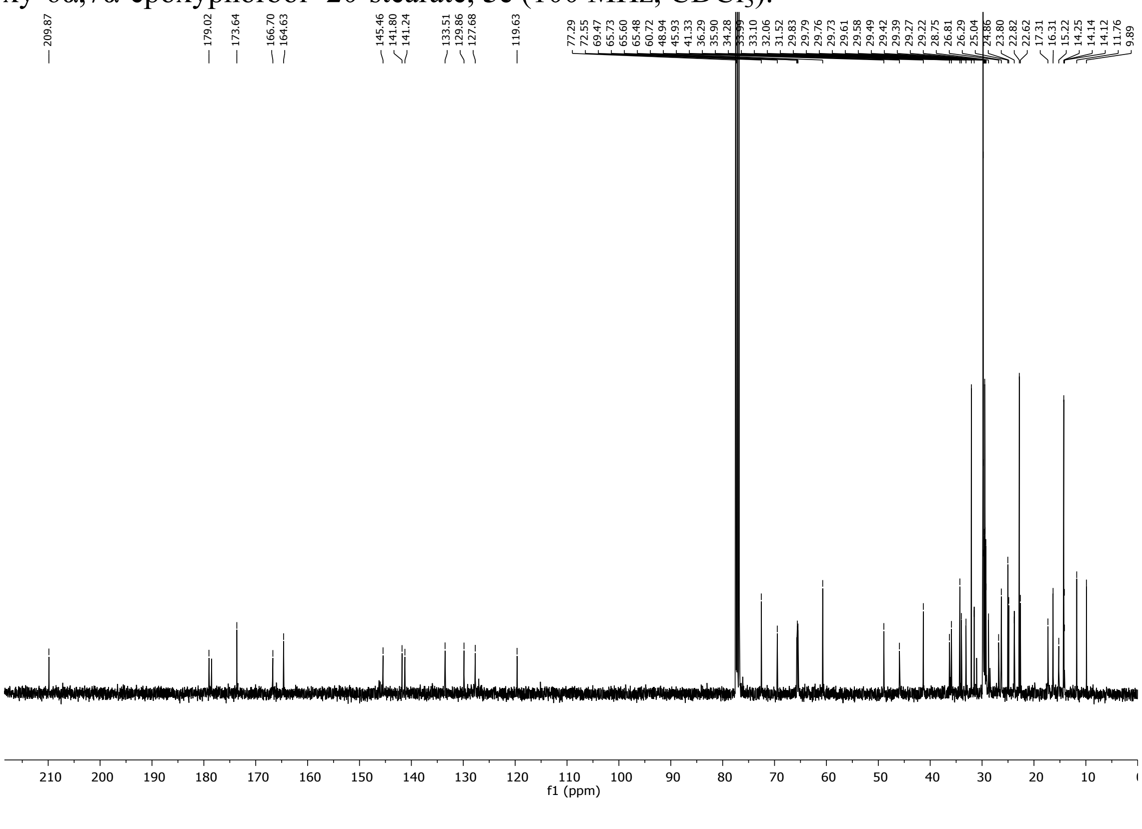

Table S2 –Summary of ^{1}H NMR data for the semisynthetic epoxytigliane triesters and related starting material (400 MHz, $CDCl_3$)

	**2a (EBC-46)**	**2b**	**2e**	**2f**	**5a (EBC-59)**	**5b**	**5c**	**5d**
	^{1}H, ppm – multiplicity – *J*, Hz							
**C-1**	7.73 dd (2.6, 1.4)	7.71 s	7.71 s	7.73 dd (2.5, 1.4)	7.73 dd (2.7, 1.3)	7.72 s	7.72 s	7.74 s
**C-2**								
**C-3**								
**C-4**								
**C-5**	4.24 s	4.26 d (2.4)	4.26 s	4.24 s	4.24 s	4.26 s	4.26 s	4.26 s
**C-6**								
**C-7**	3.28 s	3.14 s	3.14 s	3.15 s	3.28 s	3.15 (overlapped)	3.14 s	3.14 s
**C-8**	3.19 d (6.7)	3.19 d (6.5)	3.19 m	3.19 d (6.3)	3.19 d (6.7)	3.18 (overlapped)	3.19 d (6.6)	3.19 d (6.8)
**C-9**								
**C-10**	4.08 p (2.9)	4.10 t (3.0)	4.10 (overlapped)	4.09 t (2.6)	4.08 (2.7)	4.10 t (3.01)	4.11 m	4.10 m
**C-11**	1.96 dq (10.0, 6.5)	1.96 dd (9.9, 6.3)	1.96 dd (10.0, 6.4)	1.96 dd(10.1, 6.4)	1.97 dd (9.9, 6.5)	1.98 m	1.99 (overlapped)	2.01 (overlapped)
**C-12**	5.45 d (10.0)	5.44 d (9.9)	5.44 d (9.9)	5.43 d (9.9)	5.43 d (9.9)	5.41 d (10.0)	5.43 d (9.9)	5.38 (overlapped)
**C-13**								
**C-14**	1.28 d (6.8)	1.30 (overlapped)	1.28 (overlapped)	1.28 (overlapped)	1.27 (overlapped)	1.28 (overlapped)	1.28 (overlapped)	1.26 (overlapped)
**C-15**								
**C-16**	1.27 s	1.27 s	1.27 s	1.27 s	1.26 s	1.27 s	1.27 s	1.27 s
**C-17**	1.24 s	1.25 s	1.24 s	1.25 (overlapped)	1.24 s	1.25 s	1.25 s	1.25 s
**C-18**	0.87 d (6.5)	0.88 d (6.5)	0.88 d (6.8)	0.88 m	0.87 d (6.9)	0.88 d (6.8)	0.88 d (6.9)	0.90 d (6.9)
**C-19**	1.76 dd (2.9, 1.3)	1.76 d (2.5)	1.75 d (2.3)	1.78 d (2.5)	1.76 dd (2.8, 1.3)	1.76 dd (3.0, 1.3)	1.77 m	1.76 dd (2.9, 1.3)
**C-20**	3.84 AB (12.5)	4.80-3.82 AB (11.9)	4.80-3.82 AB (11.9)	4.79-3.81 AB (11.6)	3.84 AB (12.6)	4.80-3.82 AB (12.0)	4.80-3.82 AB (11.9)	4.80-3.82 AB (11.9)

The resonances of the 12- and 13-acyl moieties were very similar in all compounds. Values for **2a** and **5a** are given as representative. For **2a**: 12-Tiglate: δ 6.81 (1H, dddd, *J* = 8.5, 7.1, 5.7, 1.5 Hz, H-3'), 1.82 (3H, t, *J* = 1.3 Hz, H-5'), 1.79 (3H, dd, *J* = 7.1, 1.2 Hz, H-4'); 13-(α-methylbutyrate): δ 2.40 (1H, p, *J* = 7.0 Hz, H-2''), 1.73 (1H, m, H-3''), 1.46 (1H, tt, *J* = 14.0, 7.3 Hz, H-3''), 1.14 (3H, d, *J* = 7.0 Hz, H-5''), 0.94 (3H, t, *J* = 7.5 Hz, H-4''). **For 5a**: 12-(*E,E,E-* 2,4,6-dodecatrienoate): δ 7.25 (1H, d, *J* = 15.2, 11.2 Hz, H-3'), 6.52 (1H, dd, *J* = 15.4, 10.9 Hz, H-5'), 6.20 (1H, dd, *J* = 15.0, 11.4 Hz, H-4'), 6.12 (1H, m, H-6'), 5.92 (1H, m, H-7'), 5.81 (1H, d, *J* = 15.2 Hz, H-2'), 2.21-2.08 (2H, m, H-8'), 1.39 (2H, m, H-8'), 1.29 (2H, overlapped, H-10'), 1.26 (2H, overlapped, H-9'), 0.87 (3H, overlapped, H-12'); 13-(α-methylbutyrate): δ 2.38 (1H, sxt, *J* = 7.0 Hz, H-2''), 1.71 (1H, m, H-3''), 1.45 (1H, dd, *J* = 14.1, 7.0 Hz, H-3''), 0.92 (3H, t, *J* = 7.5 Hz, H-4''), 1.14 (3H, d, *J* = 7.0 Hz, H-5'').

Resonances of the 20-ester groups were assigned as follows:

Linoleate (values for **2b** as representative): 5.34 (4H, tt, *J* = 11.1, 5.4 Hz, H-9''', H-10''', H-12''', H-13'''), 2.76 (2H, t, *J* = 6.5 Hz, H-11'''), 2.33 (2H, t, *J* = 7.5 Hz, H-2'''), 2.04 (4H, q, *J* = 7.1 Hz, H-8''', H-14'''), 1.62 (2H, p, *J* = 7.5 Hz, H-3'''), 1.29 (14 H, overlapped, H-4''', H-5''', H-6''', H-7''', H-15''', H-16''', H-17'''), 0.94 (3H, t, *J* = 7.5 Hz, H-18'''); Oleate (values for **2c** as representative): 5.38-5.29 (2H, m, H-9''' and

H-10’’’), 2.35 (2H, t, *J* = 6.9 Hz, H-2’’’), 2.01 (4H, m, H-8’’’ and H-11’’’), 1.62 (4H, m, H-7’’’ and H-12’’’), 1.24-1.20 (18H, H-3’’’,H-4’’’, H-5’’’, H-6’’’, H-13’’’, H-14’’’, H-15’’’,H-16’’’), 0.86 (3H, overlapped, H-18’’’); Linolenate (values for **2d** as representative): 5-42-5.28 (6H, m, H-9’’’, H-10’’’, H-12’’’, H-13’’’, H-15’’’, H-16’’’), 2.84-2.77 (4H, m, H-11’’’, H-14’’’), 2.34 (2H, t, *J* = 7.5 Hz, H-2’’’), 2.06 (4H, m, H-8’’’ and H-17’’’), 1.32 (10H, overlapped, H-3’’’, H-4’’’, H-5’’’, H-6’’’, H-7’’’), 0.94 (3H, t, *J* = 7.4 Hz, H-18’’’)
